# Lack of Effective Anti-Apoptotic Activities Restricts Growth of *Parachlamydiaceae* in Insect Cells

**DOI:** 10.1371/journal.pone.0029565

**Published:** 2012-01-09

**Authors:** Barbara S. Sixt, Birgit Hiess, Lena König, Matthias Horn

**Affiliations:** Department of Microbial Ecology, University of Vienna, Vienna, Austria; University of California San Francisco, University of California, Berkeley, and the Children's Hospital Oakland Research Institute, United States of America

## Abstract

The fundamental role of programmed cell death in host defense is highlighted by the multitude of anti-apoptotic strategies evolved by various microbes, including the well-known obligate intracellular bacterial pathogens *Chlamydia trachomatis* and *Chlamydia* (*Chlamydophila*) *pneumoniae*. As inhibition of apoptosis is assumed to be essential for a successful infection of humans by these chlamydiae, we analyzed the anti-apoptotic capacity of close relatives that occur as symbionts of amoebae and might represent emerging pathogens. While *Simkania negevensis* was able to efficiently replicate within insect cells, which served as model for metazoan-derived host cells, the *Parachlamydiaceae* (*Parachlamydia acanthamoebae* and *Protochlamydia amoebophila*) displayed limited intracellular growth, yet these bacteria induced typical features of apoptotic cell death, including formation of apoptotic bodies, nuclear condensation, internucleosomal DNA fragmentation, and effector caspase activity. Induction of apoptosis was dependent on bacterial activity, but not bacterial *de novo* protein synthesis, and was detectable already at very early stages of infection. Experimental inhibition of host cell death greatly enhanced parachlamydial replication, suggesting that lack of potent anti-apoptotic activities in *Parachlamydiaceae* may represent an important factor compromising their ability to successfully infect non-protozoan hosts. These findings highlight the importance of the evolution of anti-apoptotic traits for the success of chlamydiae as pathogens of humans and animals.

## Introduction

In multicellular organisms, not only cell proliferation and differentiation, but also cell death is tightly regulated to maintain tissue homeostasis and to permit proper execution of developmental processes. Apoptosis is a major physiologic process of cell elimination [1], [2], which is mediated by an internal genetic program that can be triggered by various intrinsic or extrinsic signals. It is therefore considered as a form of “programmed cell death” (PCD) [2]. Cells dying by apoptosis undergo typical morphological and biochemical changes, including cell shrinkage, plasma membrane blebbing, nuclear chromatin condensation and fragmentation, internucleosomal DNA fragmentation, and optionally break-down of the cell into apoptotic bodies [1], [3]. Under physiological conditions, dying cells and apoptotic bodies are rapidly phagocytosed by neighboring cells or professional phagocytes [1], [4]. In contrast to accidental necrosis, which is a consequence of extreme physicochemical stress resulting in cell lysis, release of cellular contents, and inflammation [5], apoptosis is thus considered as an immunological silent form of cell death that allows an economical and safe removal of superfluous cells, avoiding detrimental effects on the whole organism [1], [6].

In accordance with the concept that the capability to undergo PCD has been a prerequisite for the evolution of multicellular life, fundamental principles of the apoptotic program are highly conserved among diverse groups of multicellular animals, including mammals and other vertebrates, but also invertebrates, such as the fruit fly *Drosophila melanogaster* and the nematode *Caenorhabditis elegans* [7], [8]. Thus, despite differences in cell death regulation [9], apoptotic signaling ultimately leads to the activation of a conserved class of proteases, the caspases, which are the main executors of the death program and whose activity results in the characteristic morphological and biochemical changes that accompany apoptotic cell death [10], [11].

In addition to its role in development and tissue homeostasis, PCD is also considered as part of the immune system of animals as it enables removal of damaged and infected cells [12]. Its protective role is underscored by the multitude of anti-apoptotic strategies used by various obligate intracellular bacteria (e.g. *Rickettsia rickettsii*, *Ehrlichia chaffeensis*) and viruses (e.g. cowpox virus, baculovirus) to overcome premature death of their replicative niche [13]–[16]. Mechanisms exploited for the manipulation of the apoptotic machinery have been exceptionally well studied for bacterial pathogens within the family *Chlamydiaceae* [17], [18], such as *Chlamydia trachomatis*, which causes trachoma and sexually transmitted disease [19], [20], and *Chlamydia* (a.k.a. *Chlamydophila*) *pneumoniae*, which is a causative agent of pneumonia but is also associated with numerous chronic diseases [21]. The more recently discovered *Parachlamydiaceae*, including *Parachlamydia acanthamoebae* and *Protochlamydia amoebophila*, as well as *Simkania negevensis* (family *Simkaniaceae*), are closely related to the *Chlamydiaceae*, with which they also share an obligate intracellular life-style that is characterized by replication within an intracellular vacuole termed inclusion, and a biphasic developmental cycle [22]. The latter consists of an alternation between two physiologically and morphologically distinct stages, the infectious elementary bodies (EBs) and the dividing reticulate bodies (RBs) [23]. Yet, the host range of these chlamydiae differs extensively. Whereas *Chlamydiaceae* are pathogens of humans and animals, the *Parachlamydiaceae* represent natural symbionts of free-living amoebae, such as *Acanthamoeba* spp. [22], [24]. *S. negevensis* displays an exceptionally broad host range, as successful infection can be observed not only in amoebae, which have been proposed to serve as natural hosts for these bacteria in the environment [25], [26], but also in epithelial and endothelial cells, as well as macrophages of human origin [27]–[29]. These versatile infection capabilities are in good agreement with the proposed role of *S. negevensis* as emerging human pathogen [27]. Although the *Parachlamydiaceae* have also been suggested to have potential impact on human health, in particular due to evidence for a possible association with lower respiratory tract infections [30]–[33], their ability to thrive within cells derived from multicellular hosts is not very well understood. However, growth of *Parachlamydiaceae* within mammalian cell culture appears to be rather limited, especially if compared to infections within their natural amoebal hosts [34]–[39]. Moreover, in some studies cytotoxic effects [36] or even features of apoptotic death [35] have been reported in mammalian cells challenged with strains of *Pa. acanthamoebae*.

Although forms of PCD have been reported to exist in some groups of protozoa, these do not operate through the canonical apoptotic machinery of metazoans [7], [40], suggesting that microbes infecting unicellular eukaryotes do not have to struggle with this sophisticated defence mechanism. Inspired by the observations reported for the mammalian host cell system we thus hypothesized that the *Parachlamydiaceae* may lack the ability to actively block apoptosis, which for the pathogenic *Chlamydiaceae* has been shown to be an essential trait for successful completion of their developmental cycle [41]. Lack of anti-apoptotic activities may therefore represent an important factor that could universally affect the ability of *Parachlam*ydiaceae to establish infections in cells derived from multicellular hosts thereby restricting their host range. In this study, we applied an invertebrate host cell system to test the validity of this hypothesis and to further explore the potential host spectrum of amoeba-associated chlamydiae. We show that while *S. negevensis* efficiently replicates in insect cells, the *Parachlamydiaceae* are only able to establish a successful infection in these cells if apoptosis is blocked experimentally. This finding indicates that the ability to interfere with the host's apoptosis pathway is a key mechanism determining host specificity of chlamydiae.

## Results

### Chlamydial symbionts of amoebae enter and replicate within insect cells

In order to further explore the infection capabilities of the *Parachlamydiaceae* and *S. negevensis* we analyzed their ability to replicate within cell lines originating from insects. Cells lines tested in this study include the phagocytic S2 cell line derived from the fruit fly *Drosophila melanogaster* [42], the Sf9 cell line, which is a clonal isolate of the IPLB-SF-21 cell line originating from the moth *Spodoptera frugiperda* [43], and the Aa23T cell line derived from the mosquito *Aedes albopictus* [44]. Bacteria, which were routinely cultivated within *Acanthamoeba* sp. UWC1, were purified from their amoebal host cells and transferred to insect cells at a defined multiplicity of infection (MOI). Addition of bacteria was followed by low speed centrifugation, which facilitates infection with chlamydiae (Fig. S1) [45]–[47], and the medium was then immediately exchanged to synchronize the infection.

**Figure 1:**
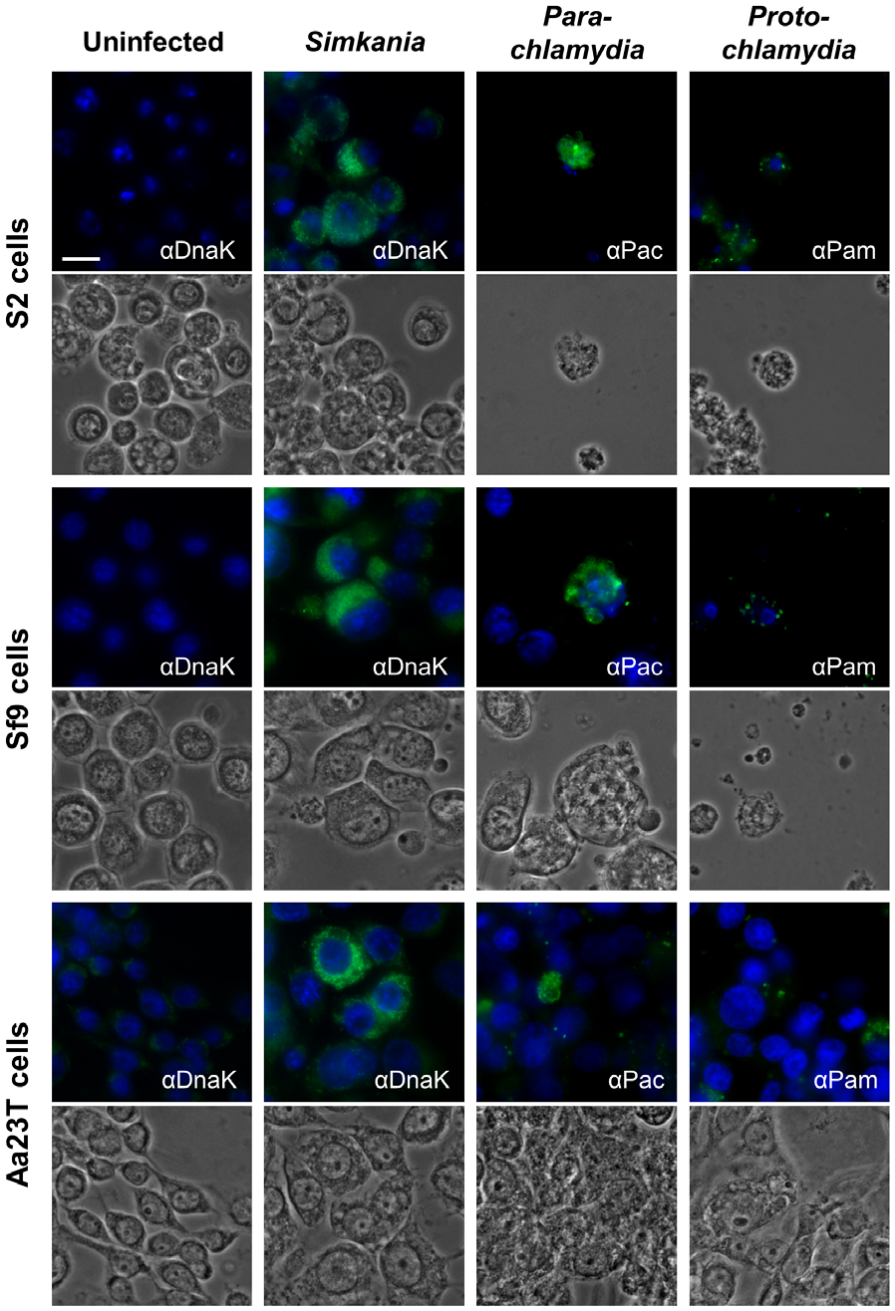
Chlamydial symbionts of amoebae enter and replicate within insect cells. The three insect cell lines S2, Sf9, and Aa23T were either left untreated or were infected with *S. negevensis* (MOI 40), *Pa. acanthamoebae* (MOI 1), or *P. amoebophila* (MOI 5). At 72 h p.i. bacteria were visualized by immunostaining (green) using antibodies raised against the protochlamydial heat-shock protein DnaK (αDnaK), purified *Pa. acanthamoebae* UV7 (αPac), or purified *P. amoebophila* UWE25 (αPam). DNA was stained with DAPI (blue). The bar corresponds to 10 µm.

**Figure 2:**
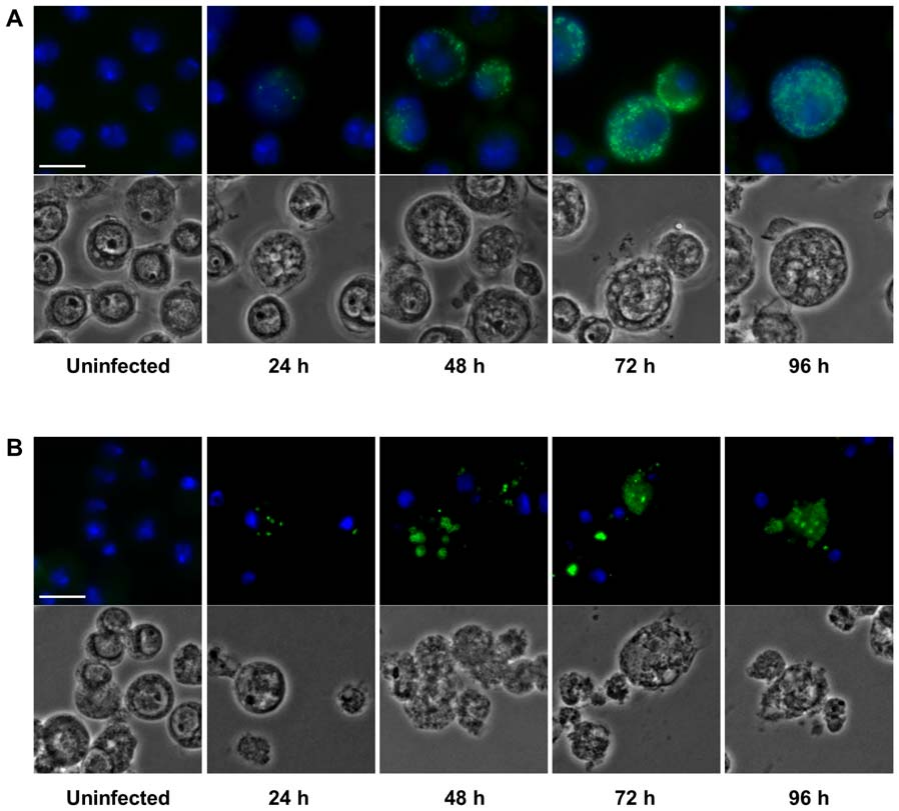
Infection cycle of *S. negevensis* (A) and *Pa. acanthamoebae* (B) in S2 cells. S2 cells were either left untreated or were infected with *S. negevensis* (MOI 5) (A) or *Pa. acanthamoebae* (MOI 0.5) (B). At indicated time points, bacteria were visualized by immunostaining (green) using antibodies raised against the protochlamydial heat-shock protein DnaK (A) or purified *Pa. acanthamoebae* UV7 (B). DNA was stained with DAPI (blue). The bar corresponds to 10 µm.

Immunostaining of bacteria revealed that *S. negevensis* was able to efficiently enter and replicate within all three tested insect cell lines (Fig. 1). Although the proportion of infected cells was variable among cell lines, in general a MOI of 40 was sufficient to cause infections in more than 50% of all cells in infected cultures. Bacterial replication was first apparent between 24 h and 48 h post infection (p.i.) (Fig. 2A and S2). At 72 h p.i. most infected cells contained already high numbers of intracellular bacteria that appeared to be localized either within a single large inclusion or numerous smaller vacuoles. Cells that harbored only small inclusions or few single bacteria were, however, also visible at late stages, suggesting that bacterial growth did not proceed equally fast in all infected cells. Cultures infected with an MOI of 20 could be maintained by regular passage for about two to three weeks, after which cell numbers stopped to increase and finally rapidly decreased due to cell lysis. At this time point, culture supernatants contained infectious particles that could establish a second round of infection after transfer to uninfected cultures (data not shown), suggesting that *S. negevensis* is able to successfully complete its infection cycle within these host cells.

*Pa. acanthamoebae* was also able to replicate within all three insect cell lines (Fig. 1), but induced severe cytotoxic effects. Bacterial growth was first evident between 24 h and 48 h p.i., when tiny inclusions appeared in a small proportion of infected cells (Fig. 2B and S2). Intracellular vacuoles then grew in size as bacterial numbers progressively increased resulting in heavily infected cells that were first detectable at 72 h p.i. Parachlamydial inclusions seemed to be more densely packed with bacteria than those formed by *S. negevensis*, and as the infection proceeded, host cell nuclei appeared to be displaced by the growing vacuoles, which could not be observed during infections with *S. negevensis*. Immunostaining of bacteria did not allow an accurate determination of the percentage of cells containing intracellular *Pa. acanthamoebae*, because microscopic inspection suggested that a significant proportion of added bacteria remained firmly attached to the extracellular surface of the cells and could not be removed by additional washing steps. Efficient bacterial growth resulting in visible inclusions occurred, however, only in very few cells within infected cultures. Although higher bacterial doses might increase the infection efficiency, relatively low numbers of bacteria (MOI 0.5 to 5) were preferentially used for *Parachlamydiaceae* due to the strong cytotoxicity observed in cultures infected with these bacteria.

Intracellular bacteria, as well as cytotoxic effects, were also observed in insect cell cultures treated with *P. amoebophila* (Fig. 1), yet formation of large inclusions could not be observed even at later stages of infection. This finding is consistent with the reported growth behavior of this strain that also in amoebae appears to form rather small inclusions, containing only single or very few bacterial cells, which can be found scattered throughout the host cell [48]. In addition, as in the case of *Pa. acanthamoebae*, complete removal of extracellular *P. amoebophila* turned out to be impossible, which further complicated monitoring of the infection progress over time.

Altogether these findings indicate that the amoeba-associated chlamydiae *P. amoebophila*, *Pa. acanthamoebae*, and S. *negevensis* have the capacity to infect cells derived from insects and that at least the latter two are also able to replicate within these invertebrate host cells.

### *Parachlamydiaceae*, but not *Simkania*, induce extensive cell death in insect cells

**Figure 3:**
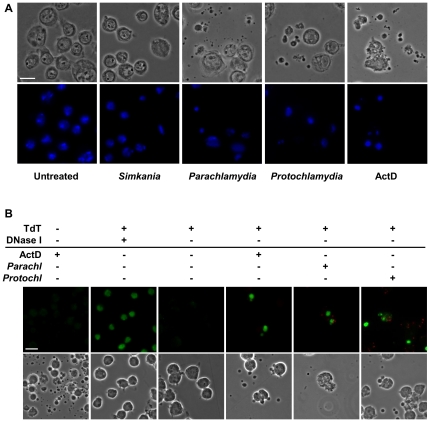
*Parachlamydiaceae*-induced cell death in S2 cells is accompanied by apoptosis-like morphological and nuclear changes and DNA fragmentation. Panel (A) illustrates morphological and nuclear changes that were observed in S2 cells in response to infection with *Parachlamydiaceae*, but not *Simkania*. Insect cells were infected with *S. negevensis*, *Pa. acanthamoebae*, or *P. amoebophila* (MOI 2.5). At 7 h p.i. DNA was stained with DAPI (blue). Untreated cells and cells treated with the apoptosis inducer ActD (7 h) are shown for comparison. The bar corresponds to 10 µm. In (B) the detection of DNA fragmentation by TUNEL staining in S2 cells infected with *Parachlamydiaceae* is shown. S2 cells were either left untreated, incubated with ActD (10 h), or infected with *Pa. acanthamoebae* or *P. amoebophila* (MOI 2.5, 10 h). Bacteria were detected by immunostaining using antibodies raised against purified bacteria (red). TUNEL-positive nuclei are shown in green. Two additional controls were included, a negative control where TdT was omitted from the TUNEL reaction mixture and a positive control where cells were preincubated with DNase I to experimentally introduce DNA double strand breaks in all (also non-apoptotic) cells. Note that apart from this control, TUNEL-positive cells typically display other characteristic features of apoptotic cells, such as condensed and fragmented nuclei and formation of apoptotic bodies. After infection, TUNEL-positive cells were also frequently associated with bacteria. The bar indicates 10 µm.

Infection of insect cells with *Parachlamydiaceae*, *Pa. acanthamoebae* or *P. amoebophila*, was accompanied by morphological changes indicative for apoptotic cell death, including cell shrinkage, cell detachment, and formation of apoptotic bodies (Fig. 3A and S3). This resulted eventually in cell lysis, which can be explained by secondary necrosis, which usually occurs *in vitro* subsequent to apoptotic cell death [49]. Moreover, these phenotypic changes were highly similar to those observed in response to actinomycin D (ActD), a known inducer of apoptotic death in insect cells [50] (Fig. 3A and S3). The extent of cell death induction in cultures infected with *Parachlamydiaceae* was dependent on the bacterial dose, yet formation of apoptotic bodies was even visible at very low MOIs, such as MOI 0.01. The time course of cellular disintegration, however, appeared to be independent of the amount of bacteria added, and morphological changes were detectable as early as 6 to 8 h p.i. Cell death did not proceed synchronously in all cells of infected cultures; its onset seemed to be delayed in some cells and few individual cells even resisted disintegration over several days. In contrast to *Parachlamydiaceae*, *S. negevensis* did not induce appreciable extents of early host cell death at comparable low MOIs (≤5) (Fig. 3A and S3), though very high bacterial doses also resulted in morphological alterations indicative for apoptotic cell death.

### *Parachlamydiaceae*-induced cell death represents caspase-dependent apoptosis and occurs early during infection

The pathogenic *Chlamydiaceae* are able to efficiently block the apoptotic machinery in infected mammalian cells [17], [18]. Although host cell death that resembles apoptosis has occasionally been reported to occur at the end of their infection cycle, this appears to be a clearly distinct mode of cellular demise, as it proceeds in the absence of caspase activation [17], [18]. We were therefore interested in a more detailed characterization of the nature of *Parachlamydiaceae*-induced cell death. In particular, we aimed to clarify whether it was accompanied by typical biochemical hallmarks of apoptotic cell death, such as changes in nuclear morphology and internucleosomal DNA fragmentation, and whether it involved activation of apoptotic effector caspases.

Nuclear morphology was analyzed microscopically after staining with the DNA dye 4′,6-diamidino-2-phenylindole (DAPI). Whereas most nuclei in untreated control cultures and cultures infected with *Simkania* displayed a normal morphology, a marked increase in condensed and fragmented nuclei, indicative for apoptotic cell death [51], was visible not only in cells treated with ActD, but also in cultures infected with *Parachlamydiaceae* (Fig. 3A and S3). Condensed nuclear fragments were detected in both dying cells and apoptotic bodies.

We next investigated whether *Parachlamydiaceae*-induced cell death was also characterized by DNA fragmentation. Electrophoretic separation of DNA isolated from Sf9 cultures that were infected with *Pa. acanthamoebae* or *P. amoebophila* revealed a DNA ladder consisting of bands that were multiplies of about 180–200 bp in size (Fig. S4). This band pattern, which is typical for apoptotic internucleosomal DNA fragmentation [52], [53], could also be observed after experimental apoptosis induction by ActD. In contrast, only high molecular weight DNA was detectable in untreated control cells. For unknown reasons, apoptotic DNA ladders could neither be detected in infected S2 cells nor in S2 cultures treated with ActD, despite detectable cell death induction. We therefore additionally applied the TUNEL (terminal deoxynucleotidyl transferase dUTP nick end labelling) assay [54], which allows *in situ* detection of DNA strand breaks in host nuclei, as alternative technique. This method not only confirmed occurrence of DNA fragmentation in infected Sf9 cultures (Fig. S5), but an increase in TUNEL-positive cells, compared to untreated cultures, was also detectable for infected S2 cells (Fig. 3B). TUNEL-staining was also observed after ActD treatment and moreover typically coincided with other features of apoptotic cell death, such as nuclear condensation and formation of apoptotic bodies that frequently also contained TUNEL-positive, condensed nuclear fragments.

**Figure 4:**
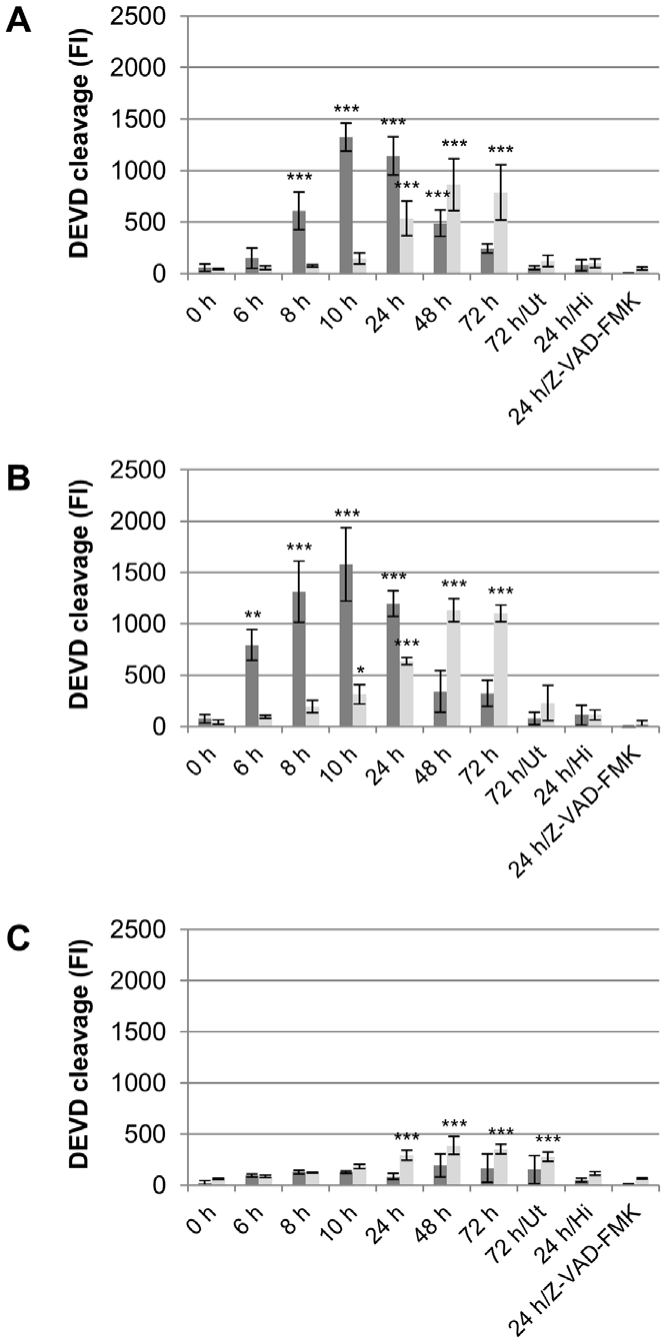
Time course of *Parachlamydiaceae*-induced effector caspase activity in S2 cells. S2 cells were infected with *Pa. acanthamoebae* (A), *P. amoebophila* (B), or *S. negevensis* (C) at a MOI of 5. Untreated cells (Ut) and cells treated with heat-inactivated bacteria (Hi) or with infectious bacteria in the presence of the pan caspase inhibitor Z-VAD-FMK (10 µM) served as controls. Activity of effector caspases in cell lysates (dark gray) and culture supernatants (light gray) was measured at indicated time points by application of an *in vitro* DEVD cleavage assay, in which substrate cleavage results in an increase in fluorescence intensity (FI). Mean values and standard deviations of four replicates are shown. Statistical significant differences compared to 0 h p.i. are indicated (ANOVA & Scheffé ; ***, p≤0.001; **, p≤0.01; *, p≤0.05). ActD-treated cells (14 h) were used as additional positive control for the assay and resulted in mean fluorescence intensities of 3261 and 658 (standard deviation 1010 and 473) in cell lysates and supernatants, respectively.

Finally, we investigated whether *Parachlamydiaceae*-induced cell death also involved activation of apoptotic effector caspases. We used an *in vitro* enzyme assay based on a fluorimetric substrate containing the peptide DEVD, which is specifically cleaved by mammalian effector caspases and homologous enzymes in insects [10], [11]. As initial experiments indicated DEVD cleavage activity in response to infection with *Parachlamydiaceae* (data not shown), we applied this assay to monitor effector caspase activity in both cell lysates and supernatants of S2 and Sf9 cultures over three consecutive days of infection with *Pa. acanthamoebae*, *P. amoebophila*, or *S. negevensis* in order to obtain a more comprehensive picture of the extent and time course of cell death induction. Compared to untreated control cells, significantly elevated cleavage activity could be observed in S2 and Sf9 cells that were either incubated with ActD or infected with *Parachlamydiaceae* (Fig. 4 and S6) (ANOVA & Scheffé, p≤0.001). Moreover, the pan caspase inhibitor Z-VAD-FMK [55] was not only able to completely inhibit DEVD cleavage, but also all other signs of cell death usually observed at early stages of infections with *Parachlamydiaceae*. Consistent with the observation that *S. negevensis* does not induce appreciable host cell death at an equivalent bacterial dose, only a slight, though statistically significant (p≤0.001), increase in DEVD cleavage activity could be observed after infection with these bacteria (Fig. 4 and S6).

An increase of DEVD cleavage activity in S2 and Sf9 cells infected with *Parachlamydiaceae* was first visible as early as 6 h or 8 h p.i., respectively. A statistically highly significant difference (p≤0.001) compared to 0 h p.i. was first detected after 8 h in S2 cells and after 24 h in Sf9 cells. DEVD cleavage activity in cell lysates initially increased over time, but was then followed by a marked decline, which most likely reflects a transition from early apoptotic to late apoptotic/secondary necrotic cells. Consistently, decreasing cleavage activity in lysates was also accompanied by an increasing activity detectable in culture supernatants, which indicates release of active caspases from secondary necrotic cells.

In conclusion, these data demonstrate that *Parachlamydiaceae*-induced cell death in insect cells is indeed of apoptotic nature and depends on effector caspase activity. The combination of DAPI and TUNEL staining with immunodetection of bacteria moreover revealed that dying cells were frequently associated with bacteria, yet cell death induction was not restricted to infected cells, but also occurred in uninfected cells within infected cultures. Consistent with our morphological observations of cell death, *Parachlamydiaceae*-induced apoptosis in insect cells starts at a very early stage of infection and *in vitro* rapidly proceeds to secondary necrosis.

### Apoptosis induction by *Parachlamydiaceae* depends on bacterial activity

As a first step to decipher how *Parachlamydiaceae* may induce apoptosis, we aimed to analyze the specific requirements for cell death induction. In particular, we were interested in the question whether only infectious bacteria had this capacity or whether cell death might in fact be caused by cytotoxic factors or bacterial cell constituents present in chlamydial preparations. In addition, we wanted to exclude that apoptosis was triggered by cell debris originating from the amoebae from which the bacteria were purified. For this purpose S2 and Sf9 cells were subjected to diverse treatments followed by monitoring of morphological changes using phase contrast microscopy and quantitative evaluation of nuclear morphology 48 h after treatment.

**Figure 5:**
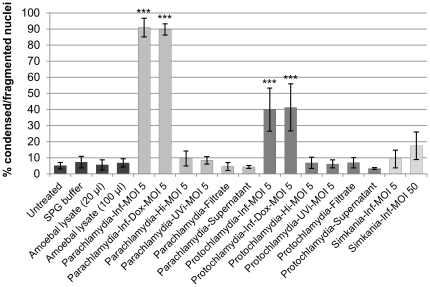
*Parachlamydiaceae*-induced changes in nuclear morphology in S2 cells depend on bacterial activity. S2 cells were either left untreated or were treated with SPG buffer, amoebal lysate, infectious *Parachlamydiaceae* (*Pa. acanthamoebae* or *P. amoebophila*; MOI 5) in the absence (Inf) or presence of the protein synthesis inhibitor doxycycline (Inf-Dox), heat-inactivated bacteria (Hi), UV- inactivated bacteria (UVi), a sterile-filtrate of the suspension of purified infectious bacteria (Filtrate) or a supernatant collected 48 h p.i. from an infected (MOI 5) apoptotic culture (Supernatant). For comparison, cells treated with infectious *S. negevensis* Z (MOI 5 or 50, as indicated) are shown. After 48 h incubation, DNA was stained with DAPI and the proportion of nuclei with altered morphology was determined. Mean values and standard deviations of six replicates (derived from three independent experiments) are shown. At least 500 nuclei per replicate were considered. Statistically significant differences compared to the untreated cells are indicated (ANOVA & Scheffé; ***, p≤0.001; **, p≤0.01; *, p≤0.05).

Based on morphological observations, cell death induction could only be detected after addition of infectious *Parachlamydiaceae* (MOI 5) and to a much lower extent also after infection with ten times higher doses of *S. negevensis* (MOI 50) (data not shown). A statistical significant increase in altered nuclei compared to untreated control cells was, however, only induced by infectious *Parachlamydiaceae* (ANOVA & Scheffé, p≤0.001), but not by *S. negevensis* (MOI 5 or 50) (Fig. 5 and S7). Significant changes in nuclear morphology could also not be observed in response to heat- or UV- inactivated *Parachlamydiaceae*, sterile filtrates of suspensions of purified infectious bacteria or lysates of *Acanthamoeba* sp. UWC1 (p>0.05), indicating that apoptosis induction depends on viable, infectious *Parachlamydiaceae*. However, *de novo* bacterial protein synthesis was dispensable, as induction of cell death was unaffected by doxycycline, an inhibitor of bacterial translation. Doxycycline was applied at a concentration above the minimal inhibitory concentration (MIC) reported for *Pa. acanthamoebae* strains Bn_9_ and Hall's coccus [34], which in our study additionally appeared to be nontoxic for insect cells, but sufficient to effectively block growth, but not entry, of *Pa. acanthamoebae* in S2 cells (data not shown). Moreover, supernatants collected 48 h after infection with *Parachlamydiaceae* from clearly apoptotic cultures did not induce significant nuclear alterations after transfer to uninfected cultures (p>0.05), suggesting the absence of cell death stimulating factors in culture supernatants at this time (Fig. 5 and S7).

Consistent with these findings, heat- and UV-inactivated *Parachlamydiaceae* did not trigger internucleosomal DNA fragmentation as indicated by absence of apoptotic DNA ladders in treated Sf9 cells (Fig. S4). Moreover, in response to heat-inactivated bacteria no increase in effector caspase activity was detected in S2 and Sf9 cells (Fig. 4 and S6). Together these results strongly suggest that apoptosis induction by *Parachlamydiaceae* requires the activity of infectious bacteria, but is independent of *de novo* synthesis of bacterial proteins.

### Experimental inhibition of apoptosis enhances infection with *Parachlamydiaceae*

The time course of apoptosis induction by *Parachlamydiaceae* suggested that the infection progress might be severely compromised due to an early disruption of chlamydial development. In order to test this hypothesis, we undertook a quantitative comparison of infections of S2 and Sf9 cells with *Pa. acanthamoebae* or *S. negevensis* in the absence or presence of the pan caspase inhibitor Z-VAD-FMK. At ten time points during a total period of 14 days the percentage of infected cells and the number of intracellular bacteria per cell were determined. To achieve an accurate quantification, which should be less affected by extracellularly attached bacteria compared to immunostaining, fluorescence *in situ* hybridization (FISH) targeting ribosomal rRNAs [56], [57] was used for the detection of bacteria. Although FISH may lead to an underestimation of the infection at early stages, its ability to predominately detect metabolically highly active bacteria, compared to inactive or dead bacteria [57], was considered as major advantage of this technique.

**Figure 6:**
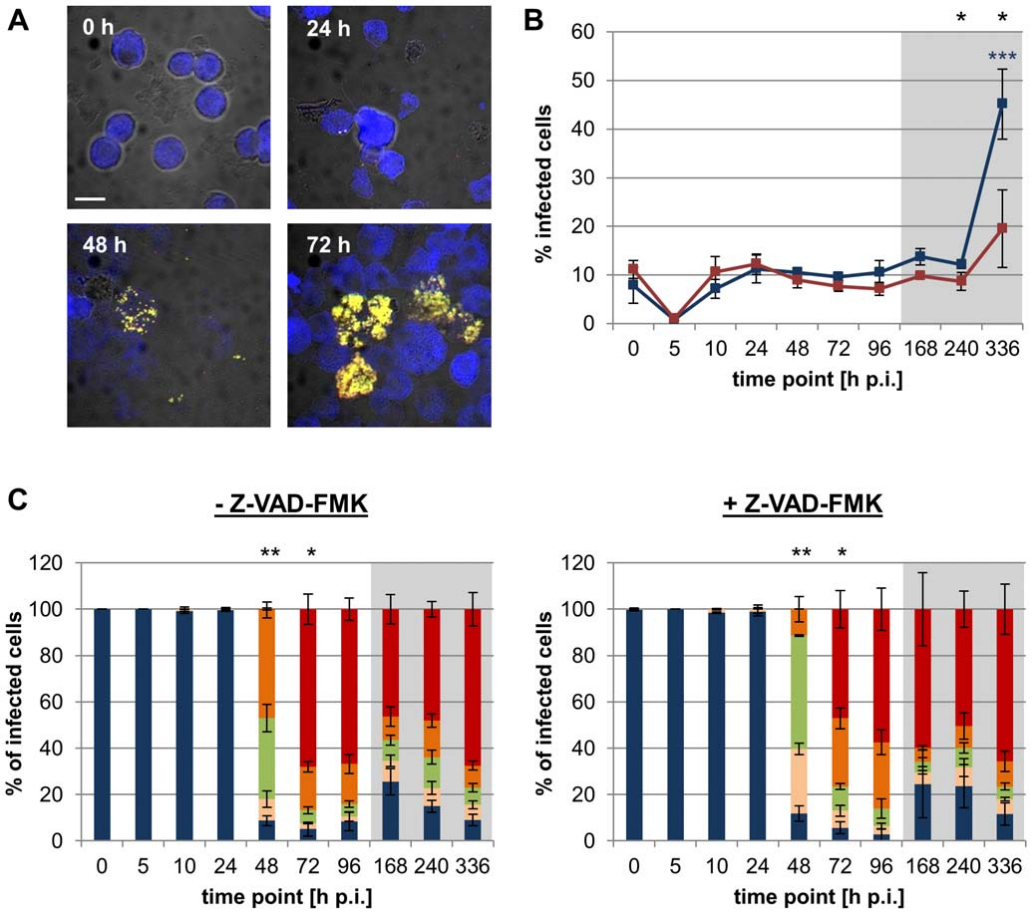
Effect of caspase inhibition on the infection of S2 cells with *S. negevensis*. S2 cells were infected with *S. negevensis* (MOI 5) and were incubated for the indicated time periods in the absence or presence of the pan caspase inhibitor Z-VAD-FMK (10 µM). Bacteria were detected with the FISH probes Simneg183 (Fluos, green) and Chls-0523 (Cy3, red), host cells with the probe EUK516 (Cy5, blue). Representative confocal images of the infection cycle (in the absence of Z-VAD-FMK) are shown in (A). The bar indicates 10 µm. The percentage of infected cells observed in the absence (blue) or presence (red) of Z-VAD-FMK over the course of infection is depicted in (B). Black stars indicate statistically significant differences between both curves (t-test) and colored stars indicate significant differences to the respective 0 h p.i. time point (ANOVA & Scheffé). Numbers of intracellular bacteria per infected cell were determined and are shown in (C). Infected cells were classified into 5 groups according to the number of intracellular bacteria (1–3, blue; 4–10, rose; 11–30, green; 31–100, orange; >100, red). Stars indicate statistically different distributions among these classes at a given time point between infections that occurred in the absence or presence of Z-VAD-FMK (χ^2^ test). In (B) and (C) mean values and standard deviations of three replicates are shown (***, p≤0.001; **, p≤0.01; *, p≤0.05). The gray boxes indicate time points that were analyzed after cells had been passaged.

Consistent with our earlier results (Fig. 2A and S2) intracellular replication of *S. negevensis* in absence of Z-VAD-FMK was not detectable in S2 and Sf9 cells within the first 24 h, after which bacterial numbers in infected cells, however, increased (Fig. 6 and S8). Starting from 72 h p.i., when cells that were densely filled with bacteria first appeared, cells reflecting variable stages of infections were visible throughout the two weeks of analysis. In the presence of Z-VAD-FMK, infections with *S. negevensis* proceeded similarly, although the growth in S2 cells appeared to be slightly slowed down. At the end of the analysis period a significant increase in the proportion of infected S2 cells compared to 0 h p.i. (ANOVA & Scheffé, p≤0.001), probably indicating release of infectious progeny and onset of the second round of infection, was visible in the absence of Z-VAD-FMK. Interestingly, although the same trend was also detectable when the caspase inhibitor was present, the increase in the percentage of infected cells was much smaller and in fact turned out to be not statistically significant (p>0.05). In case of the Sf9 cells, we did not observe a marked increase in the proportion of infected cells during the analysis period, which may indicate inefficient bacterial spread or may be explained by the fact that bacterial growth appeared to be in general delayed in this cell line.

**Figure 7:**
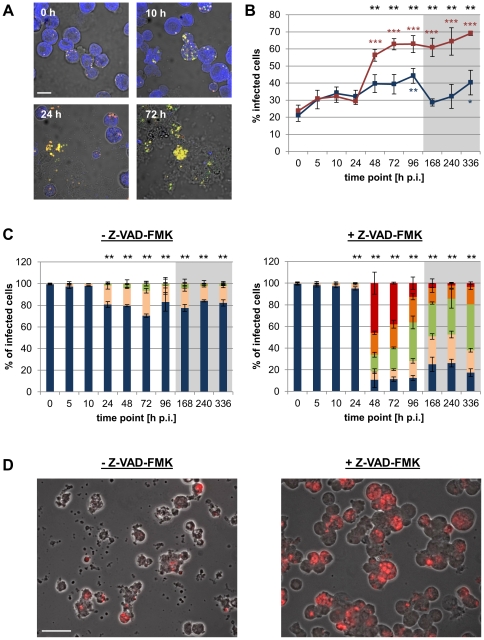
Effect of caspase inhibition on the infection of S2 cells with *Pa. acanthamoebae*. S2 cells were infected with *Pa. acanthamoebae* (MOI 5) and were incubated for the indicated time periods in the absence or presence of the pan caspase inhibitor Z-VAD-FMK (10 µM). Bacteria were detected with the FISH probes UV7-763 (Cy3, red) and Chls-0523 (Fluos, green), host cells with the probe EUK516 (Cy5, blue). Representative confocal images of the infection cycle (in the absence of Z-VAD-FMK) are shown in (A). The bar indicates 10 µm. The percentage of infected cells observed in the absence (blue) or presence (red) of Z-VAD-FMK over the course of infection is depicted in (B). Black stars indicate statistically significant differences between both curves (t-test) and colored stars indicate significant differences to the respective 0 h p.i. time point (ANOVA & Scheffé). Numbers of intracellular bacteria per infected cell were determined and are shown in (C). Infected cells were classified into 5 groups according to the number of intracellular bacteria (1–3, blue; 4–10, rose; 11–30, green; 31–100, orange; >100, red). Stars indicate statistically different distributions among these classes at a given time point between infections carried out in the absence or presence of Z-VAD-FMK (χ^2^ test). In (B) and (C) mean values and standard deviations of three replicates are shown (***, p≤0.001; **, p≤0.01; *, p≤0.05). The gray boxes indicate time points that were analyzed after cells had been passaged. The microscopic images in (D) illustrate the enhanced infection of S2 cells with *Pa. acanthamoebae* in presence of Z-VAD-FMK at 48 h p.i. Bacteria were detected with the FISH probe UV7-763 (Cy3, red). The bar corresponds to 20 µm.

In the absence of Z-VAD-FMK, infections with *Pa. acanthamoebae* were inefficient in both cell lines, as most infected cells contained only few intracellular bacteria throughout the two weeks of analysis (Fig. 7 and S8). After 24 h increased numbers of bacteria were detectable in a small proportion of infected cells, indicating the onset of bacterial replication. Yet, heavily infected cells (containing more than 100 bacteria) were hardly observed and constituted less than 1% of the infected cells at all time points analyzed. Interestingly, a slight but continuous increase in the percentage of infected cells over time was, nevertheless, observed in both cell lines. This may, however, be explained by delayed entry of bacteria that originally remained attached after the initial inoculation or by engulfment of infected apoptotic cells by neighboring cells.

In S2 cells infections with *Pa. acanthamoebae* were greatly enhanced by addition of Z-VAD-FMK (Fig. 7). Even though the initial steps including attachment and entry appeared to be unaffected by the treatment, bacterial growth, which was first detectable in few infected cells at 24 h p.i., occurred very rapidly under conditions of apoptosis inhibition. After 48 h, about 45% of the infected cells contained already large inclusions, hence the infection progress was significantly improved compared to infections that occurred in the absence of the caspase inhibitor (χ^2^ test, p≤0.01). Moreover, a strong increase in the percentage of infected cells, which was also accompanied by the onset of detectable cell lysis, was already visible at this early time point. Although infections with *Pa. acanthamoebae* in Sf9 cells were also significantly improved by caspase inhibition (Fig. S8), reinfection appeared to be less efficient in this cell line, which after an initial increase even resulted in a drop in the percentage of infected cells at later time points, possibly due to dilution effects introduced by passaging.

At the ultrastructural level, infection of apparently intact S2 cells with *Parachlamydiaceae* – including *Pa. acanthamoebae*, but also *P. amoebophila* – could be observed in the presence of Z-VAD-FMK, whereas almost all cells appeared to be in a late stage of cellular disintegration after 2 days of infection in the absence of the caspase inhibitor (Fig. S9).

In conclusion, whereas the growth of *S. negevensis* in insect cells was largely unaffected by the caspase inhibitor Z-VAD-FMK, experimental apoptosis inhibition strongly enhanced infections with *Pa. acanthamoebae*, most likely as a result of the protection of their replicative niche.

## Discussion

Chlamydiae show a broad host range and pronouncedly different life styles ranging from ubiquitous symbionts of amoebae to highly successful pathogens of humans and animals. This raises the question which underlying molecular mechanisms for host cell interaction are responsible for these differences. In this study we present evidence based on an invertebrate host cell system that the ability to block apoptosis is a key trait required for the successful infection of metazoans.

We showed that *S. negevensis*, which is known to infect both protozoa and mammalian cells [25], [26], [28], [29], is also able to replicate within insect cells. This was demonstrated by application of two different fluorescence-based detection techniques, immunostaining and FISH, coupled to conventional as well as confocal fluorescence microscopy (Fig. 1, 2A, 6, S2, and S8). Interestingly, although insect cells containing large amounts of intracellular bacteria could be observed as early as three days post infection, cell lysis and reinfection were not evident during the first 10 days of infection. A similarly long infection cycle consisting of a short exponential growth phase followed by a long plateau phase devoid of significant release of infectious progeny has been reported for *S. negevensis* in Vero cells grown at 37°C [58]–[60]. We thus exclude that our observation can be solely explained by the lower incubation temperature applied for insect cell culture (27°C). This delayed infection cycle of *S. negevensis* contrasts with the duration of those of members of the *Chlamydiaceae* and *Parachlamydiaceae*, which are typically completed within 2–5 days within their mammalian or amoebal host cells, respectively [61], [62]. Our observations suggest that the extended developmental cycle of *S. negevensis* may represent a general feature of these chlamydiae.

*Drosophila* S2 cells are an excellent model for the discovery of host factors involved in host-pathogen interactions through RNA interference based screens [63]–[65], and the ability of *S. negevensis* to thrive within insect cells may therefore be of great value for future studies. It, however, also raises the question whether an association of chlamydiae with arthropods might be in fact more widespread in the environment than known so far. Indeed several chlamydial species that are closely related to *S. negevensis*, such as *Fritschea bemisiae* and *Fritschea eriococci* (family *Simkaniaceae*) [66] as well as *Rhabdochlamydia crassificans* and *Rhabdochlamydia porcellionis* (family *Rhabdochlamydiaceae*) [67], [68], have been described to naturally infect insects or other arthropods. Moreover, S2 cells were shown to support growth of the well-known chlamydial pathogens *C. trachomatis* [69] and *C. caviae* [63], although formation of infectious progeny within these cells has so far only been reported for the latter of these two species and, as for *S. negevensis*, their capability to infect living insects remains to be elucidated.

The *Parachlamydiaceae* are naturally associated with amoebae but, in contrast to *S. negevensis*, do not efficiently thrive within mammalian cells [34]–[39]. Consistent with this notion, although *Parachlamydiaceae* are able to invade insect cells, they rapidly triggered cell death, whose apoptotic nature was inferred from the following observations: (i) phase contrast and fluorescence microscopy revealed that dying cells displayed morphological and nuclear changes indicative for apoptotic cell death [1], [51] (Fig. 3A, 5, S3, and S7); (ii) DNA fragmentation could be detected by two independent techniques, including visualization of nucleosomal DNA ladders on agarose gels [52] and the TUNEL method [54] (Fig. 3B, S4, and S5); (iii) an *in vitro* enzyme assay revealed cleavage activity towards a peptide substrate containing DEVD (Fig. 4, and S6), which is specifically cleaved by apoptotic effector caspases [10], [11]; and (iv) cell death could be completely blocked by the pan-caspase inhibitor Z-VAD-FMK (Fig. 4 and S6).

In animals, apoptotic death of infected cells is regarded as defense mechanism, which may in particular be highly effective in the battle against intracellular pathogens that rely on an intact host cell [13], [70]. This concept is well exemplified by infections with viruses such as the baculovirus *Autographa californica*, for which it has been demonstrated that disruption of a viral anti-apoptotic gene significantly reduces not only viral replication in insect cell culture, but also infectivity in *Spodoptera frugiperda* larvae [71], [72]. Likewise, *Chlamydiaceae* actively block apoptotic cell death in infected mammalian cells [73]–[77], and a very recent report described the inhibition of cell death by *S. negevensis* in human cells [78]. Experimental induction of premature host cell death has been reported to completely disrupt development of *C. trachomatis*, preventing formation of infectious progeny [41]. Maintenance of host cell viability for prolonged periods of time has also been suggested to be important during persistent chlamydial infections [79]. At late stages of infection, however, intracellular pathogens such as chlamydiae may also benefit from apoptotic host cell death, as engulfment of bacteria-loaded apoptotic bodies by neighboring cells may facilitate pathogen spread [17], [80]. Indeed it has been repeatedly reported that at the end of their infection cycle also *Chlamydiaceae* induce host cell death that shares some features with apoptosis, including externalization of phosphatidylserine that may enhance phagocytic uptake of dying cells [80], but which is clearly distinct from the apoptotic program as numerous studies failed to detect any involvement of apoptotic effector caspases [81]–[84]. However, even though the interaction of the *Chlamydiaceae* with the host cell's suicide machinery has been subject to detailed investigation during the last decades, the molecular mechanisms underlying chlamydial death-stimulating activities are still poorly understood. In addition, the significance of this apoptosis-like cell death in the context of infection and pathogen spread remains obscure. Host cell lysis and extrusion of bacteria-loaded vesicles have recently been suggested to represent the main mechanisms of host cell exit by *Chlamydiaceae* [85].

Nevertheless, the *Chlamydiaceae* have been convincingly shown to exploit a whole battery of strategies to prevent apoptotic death in mammalian host cells [17], [18]. The fact that *C. trachomatis* and *C. caviae* were shown to grow in *Drosophila* S2 cells without induction of apoptosis [63], [69] suggests that these bacteria may as well have the capacity to block cell death in these invertebrates. However, despite of the evolutionary conservation of the apoptotic core machinery, apoptosis regulation differs significantly between mammals and insects [9]. In mammals, mitochondrial outer membrane permeabilization resulting in the release of factors required for caspase activation is a central step in most apoptotic pathways and tightly regulated by the balance of anti- and pro-apoptotic proteins [86]. *Chlamydiaceae* interfere with this balance in infected cells by diverse mechanisms including degradation of the pro-apoptotic BH3-only proteins [87]–[89] and upregulation of anti-apoptotic proteins, such as Mcl-1 [90]–[92], which together confer a block of the apoptotic program at a pre-mitochondrial step. Yet in insects, although still controversial, it is assumed that caspase activation occurs independently from mitochondrial factors and that the IAP (inhibitor of apoptosis) proteins, which can directly inhibit caspases, instead play a central role in the regulation of apoptosis [93]. Interestingly, *Chlamydiaceae* – and very recently also *S. negevensis* – have been shown to trigger up-regulation of IAPs in infected mammalian cells [78], [94], [95], and a similar strategy if operational may be a highly effective mechanism to block cell death in insects as well.

In case of the *Parachlamydiaceae*, the observation that the pan caspase inhibitor Z-VAD-FMK greatly improved growth of *Pa. acanthamoebae* in insect cells (Fig. 7) clearly illustrates that premature host cell death is highly disadvantageous for these bacteria. This is consistent with the very early time point of apoptosis induction (Fig. 4 and S6), resulting in disruption of intracellular parachlamydial development. Although infections with *P. amoebophila* were not quantitatively analyzed in this study, we observed that early host cell death induced by these *Parachlamydiaceae* could also be completely blocked by the pan caspase inhibitor, and apparently intact infected cells were readily visible under such conditions. Beside its inhibitory action on caspases, Z-VAD-FMK has also been shown to affect the activity of certain lysosomal proteases, such as cathepsins [96], [97]. Given that, at least in human macrophages, *Pa. acanthamoebae* has been reported to not escape from the endocytic pathway – as observed for other chlamydial species [98], [99] – but to reside within vacuoles that acquire markers of late endosomes and even acidify [99], [100], it is conceivable that impaired lysosomal function may provide an additional benefit for the intracellular growth of *Pa. acanthamoebae*. Yet, we could observe that the pan caspase inhibitor Q-VD-OPH, which is considered to be less cross-reactive with lysosomal proteases than Z-VAD-FMK [101], has an equal capacity to enhance parachlamydial growth in insect cells (data not shown), which strongly indicates that the growth-promoting activity of Z-VAD-FMK was indeed due to the inhibition of the canonical apoptotic machinery. In view of this finding it may be an interesting task of future investigations to elucidate whether co-infections with other intracellular pathogens, which effectively antagonize the host's apoptotic response, may have a similar impact on the outcome of infections with *Parachlamydiaceae*.

How *Parachlamydiaceae* induce cell death in insect cells remains so far unknown. It has been reported that exposure to cell constituents of *Chlamydiaceae*, such as lipopolysaccharide or heat shock proteins, could trigger apoptosis in certain cell types [102]–[104]. In addition, due to the known cytotoxicity of *Acanthamoeba* spp. [105], we initially suspected that amoebal cell debris, and not the bacteria themselves, could be responsible for the observed effect. However, as inactivated bacteria, filtrates of bacterial suspensions, and lysates of *Acanthamoeba* sp. all failed to induce any signs of apoptotic cell death (Fig. 4, 5, S4, S6 and S7), we ruled out these possibilities. Infections with extremely high doses of *Chlamydiaceae* were shown to cause a necrotic type of death in mammalian cells, which is characterized by cellular disintegration starting immediately after challenge with the bacteria. It has been hypothesized that this so-called “immediate cytotoxicity” of chlamydiae may be due to physical damage caused by the ingestion of high amounts of bacterial particles [106]–[108]. We exclude that *Parachlamydiaceae*-induced cell death in insect cells can be explained by this phenomenon because of (i) the moderate to low MOIs applied in our study, (ii) the particular timing of cell death which is pronouncedly delayed compared to “immediate cytotoxicity”, and (iii) the observed apoptotic features. In the case of the pathogenic *Chlamydiaceae*, it has also been hypothesized that either the chlamydiae or chlamydia-infected cells may secrete death-stimulating factors, such as toxins or cytokines, into the culture supernatant, based on the observation that apoptosis was frequently observed in uninfected cells within infected cultures [81], [82], [109], [110]. Although in our study the combination of DAPI and TUNEL staining with immunodetection of bacteria also suggested that cell death induction was not exclusively restricted to infected cells, we did not observe an apoptosis-stimulating activity in supernatants of infected clearly apoptotic cultures (Fig. 5 and S7), indicating that such a factor does either not exist or must be highly unstable and only transiently detectable. Consistent with observations from infections of S2 cells with *C. caviae* [63] an alternative explanation for apoptosis induction in uninfected cells would be that cells that appeared to be uninfected at the time of analysis had originally been invaded by bacteria that were subsequently cleared by lysosomal degradation. It is also conceivable that apoptosis induction is independent of bacterial entry. Transient contact, including bacterial attachment and possibly even secretion of bacterial effector molecules into the host cell, might already be sufficient to trigger the apoptotic program. However, the fact that apoptosis induction was not affected by the bacterial protein synthesis inhibitor doxycycline (Fig. 5 and S7) indicates that bacterial factors involved in host cell killing already exist preformed in the bacteria present in the inoculum.

Although the *Parachlamydiaceae* lack genes encoding homologues of known chlamydial toxins, such as CADD (*Chlamydia* protein associating with death domains) [111] and proteins related to clostridial cytotoxins [112], their genomes encode numerous proteins of unknown function [113]–[115], and the presence of yet unrecognized toxin genes can therefore not be excluded. Alternative to the production of a genuine toxin, it is, however, equally well conceivable that the apoptotic program is triggered more indirectly through disturbance of host cell functions, which might be sensed by host cells through general stress sensors. Such an intrinsic pro-apoptotic action has also been attributed to the *Chlamydiaceae*, which, besides the need to protect mammalian host cells from effector T cell mediated killing [116], [117], has been suggested to have been a major driving force for the evolution of effective counteracting strategies [13]. In view of this perspective, it may be possible that the extant amoeba-associated *Parachlamydiaceae*, in addition to the lack of critical anti-apoptotic activities, may also have retained death-stimulating factors, which have been lost in other chlamydiae during the adaptation to multicellular hosts.

In summary, we present evidence that apoptosis is an effective host defense mechanism against *Parachlamydiaceae* in insect cells. Reports about cytotoxicity and apoptosis induction by *Parachlamydiaceae* in mammalian cell culture suggest that this concept is not restricted to insect hosts, but represents a more universal phenomenon across potential multicellular hosts of *Parachlamydiaceae*. We therefore conclude that the development of the ability to block apoptosis was a key step during the evolution of the *Chlamydiae*, which facilitated the transition from protozoa to metazoan hosts.

## Materials and Methods

### Cell culture

The insect cell lines S2 (*Drosophila melanogaster*) [42] and Aa23T (*Aedes albopictus*) [44] were maintained at 27°C in Schneider's insect medium (Sigma-Aldrich, Austria) supplemented with 10% heat-inactivated (56°C, 30 min) fetal bovine serum (PAA Laboratories, Austria). The insect cell line Sf9 (*Spodoptera frugiperda*) [43] was maintained at 27°C in Grace's insect medium (Sigma-Aldrich) supplemented with 10% fetal bovine serum (PAA Laboratories) and 2 mM L-glutamine (Sigma-Aldrich). Cells were regularly passaged at confluency. Insect cell cultures were shown to be free of contamination with *Mycoplasma* spp. by using the Venor GeM PCR kit (BioProducts, Austria). *Acanthamoeba* sp. UWC1 containing the chlamydial symbionts *P. amoebophila* UWE25 [48], *Pa. acanthamoebae* UV7 [36], or *S. negevensis* Z [60], [118] and symbiont-free isogenic amoebae were maintained at 20°C in TSY medium (30 g/l trypticase soy broth, 10 g/l yeast extract).

### Purification of chlamydial symbionts

Amoebae containing chlamydial symbionts were harvested at 3200× g and washed with Page's amoebic saline (PAS; 0.12 g/l NaCl, 0.004 g/l MgSO_4_×7H_2_O, 0.004 g/l CaCl_2_×2H_2_O, 0.142 g/l Na_2_HPO_4_, 0.136 g/l KH_2_PO_4_). Cells were resuspended in a small volume PAS and disrupted by three consecutive cycles consisting of freezing (dry ice/ethanol bath) and thawing (37°C). Then 0.5 volumes sterile glass beads (0.75–1.00 mm) were added, followed by vortexing for 3 min and centrifugation (620× g, 10 min). The supernatant was filtered (1.2 µm) and bacteria were collected by centrifugation (35 700×g, 45 min) in an Optima™ L-100 XP ultracentrifuge equipped with SW41 Ti rotor (Beckman Instruments, USA). Bacteria were resuspended in a small volume of sucrose-phosphate-glutamate (SPG) buffer (75 g/l sucrose, 0.52 g/l KH_2_PO_4_, 1.53 g/l Na_2_HPO_4_×2H_2_O, 0.75 g/l glutamic acid) and the suspension was homogenized using needles (diameter 0.90 mm and 0.45 mm). Bacteria were collected by centrifugation (as described above), resuspended in SPG, and homogenized. Aliquots of purified bacteria were stored at −80°C. If not stated otherwise, all steps during the purification were carried out at 4°C. Number of bacterial particles were determined by counting of bacteria, which were filtered onto a 0.2 µm filter (Milipore, Austria) and stained with 4′,6-diamidino-2-phenylindole (DAPI), at an epifluorescence microscope (Axioplan 2 imaging; Zeiss, Germany). To verify the identity of the chlamydiae, DNA was extracted from purified bacteria using the DNeasy blood & tissue kit (Qiagen, Austria) and the chlamydial 16S rRNA gene was amplified using a pan-chlamydia primer set described elsewhere [119]. PCR products were sequenced on an ABI 3130 XL genetic analyzer (Applied Biosystems, Austria) and compared to sequence data available at NCBI.

### Infection procedure and apoptosis induction

Insect cells seeded into 24-well plates (Nunc; 5×10^5^ cells per well), 12-well plates (Nunc; 1×10^6^ cells per well), 8-well Lab Tek™ chamberslides (Nunc; 1×10^5^ cells per well), or culture flasks (Iwaki, 25 cm^2^; 6×10^6^ cells) were infected using a defined MOI. For microscopic analyses, infections were performed on cells seeded either in chamberslides or on glass coverslips in 24-well plates. Addition of the bacteria was followed by centrifugation (130× g, 15 min, 23°C), exchange of growth medium, and incubation at 27°C until analysis. If indicated apoptosis was induced by addition of 2 µg/ml actinomycin D (ActD; Sigma-Aldrich) to the growth medium followed by incubation for indicated periods of time.

### Immunostaining and DAPI staining

Insect cells were fixed with 100% methanol (ice-cold, 5 min), washed once with phosphate-buffered saline (PBS), and blocked for 20 min in 2% bovine serum albumin (BSA) in PBS. Cells were then incubated for 1 h with primary antibodies diluted in blocking solution. Primary antibodies used in this study include polyclonal antibodies (Eurogentec, Belgium) raised against *P. amoebophila* UWE25 or *Pa. acanthamoebae* UV7, which were purified on gastrografin gradients [120], or against purified recombinant protochlamydial heat shock protein DnaK (pc1499). Antisera were pre-adsorbed with host proteins as described recently [120]. After three washing steps in PBS, cells were incubated for 1 h with Cy2- or Cy3-labelled secondary antibodies (Dianova, Germany) diluted in blocking solution, followed by three additional washing steps. DNA was stained for 10 min with DAPI (100 ng/ml in PBS). Cells were then washed twice with PBS and embedded in Mowiol [120]. Images were taken with a CCD camera (AxioCam HRc; Zeiss) connected to an epifluorescence microscope (Axioplan 2 imaging; Zeiss).

### Infection analysis by FISH

Insect cells seeded in 24-well plates were infected (as described above) and the pan-caspase inhibitor Z-VAD-FMK (10 µM; Promega, USA) was added to half of the wells, but was omitted in the others. Infections were analyzed at 0, 5, 10, 24, 48, 72, and 96 h p.i., however at 48 h p.i. the caspase inhibitor was replenished in the respective remaining wells to assure efficient caspase inhibition. In order to enable analysis of later time points (7, 10, and 14 days p.i.), cells from additional wells were passaged (1∶3) into new wells at 72 h p.i. At the time points indicated, cells were fixed by addition of one volume of a 4% formaldehyde solution to the growth medium, followed by incubation for 1 h at room temperature. Cells were then washed once with PBS and dehydrated by incubation in increasing concentrations of ethanol (50%, 80% and 96%, 3 min incubation with each). Hybridizations were performed for 3 h at 46°C at a formamide concentration of 25%, using hybridization and wash buffers described elsewhere [121]. The following fluorescently labelled (Cy3, Cy5, or Fluos) probes (Thermo Fisher Scientific, Germany) were applied in this study: EUK-516 (5′- ACCAGACTTGCCCTCC -3′) [122] for the detection of eukaryotic host cells, Chls0523 (5′-CCTCCGTATTACCGCAGC-3′) in combination with the competitor probe (5′-CCTCCGTATTACCGCGGC-3′) [56] as general probe for the detection of all chlamydiae used in this study, and UV7-763 (5′-TGCTCCCCCTTGCTTTCG-3′) [36] and Simneg183 (5′-CAGGCTACCCCAGCTC-3′) for the specific detection of *Pa. acanthamoebae* UV7 and *S. negevensis* Z, respectively. Cells were embedded in Mowiol and images were taken at the epifluorescence microscope and at a confocal laser scanning microscope (LSM 510 Meta; Zeiss). The percentage of infected cells and the number of intracellular bacteria within infected cells were determined. Both normal and dying cells were considered. For each time point and condition, data from three replicate infections were collected. In general, at least 500 cells were counted per sample. However, in case of infections of S2 cells with *Pa. acanthamoebae* UV7 in presence of Z-VAD-FMK, only a lower number of cells (at least 200 cells per well) could be analyzed for some replicates of later time points (7, 10, and 14 days p.i.) due to highly reduced cell numbers caused by cell lysis.

### Evaluation of nuclear morphology

To test for the requirement of bacterial activity for cell death induction, insect cells seeded in 24-well plates were either treated with infectious bacteria (in the presence or absence of 10 µg/ml doxycycline) or with an equivalent amount of SPG buffer or heat-inactivated (56°C, 30 min) or UV-inactivated (Transilluminator UST-30M-8E (Biostep, Germany), 15 min) bacteria, three times the amount of a sterile-filtrate (0.2 µm) of the suspension of purified bacteria, or different amounts of an amoebal lysate (20 µl or 100 µl). Centrifugation and medium exchange were carried out as described for the standard infection procedure, yet these steps were omitted in wells where 100 µl amoebal lysate were added. In an additional experiment, the growth medium of hitherto untreated cells was replaced by sterile-filtered supernatant of an infected culture, collected 48 h p.i. For the preparation of amoebal lysates, four culture flasks (25 cm^2^) containing *Acanthamoeba* sp. UWC1 (in total approximately 2×10^7^ cells) were harvested (3200× g, 10 min). Amoebae were resuspended in 1 ml PBS and lysed by 3× freeze-and thaw (see above). The lysate was filtered (1.2 µm) and stored at −80°C. The efficiency of heat- and UV-inactivation of bacteria was tested by infection of amoebae, which revealed strongly decreased, but not completely abolished, infectivity of inactivated bacteria (data not shown). After incubation for 48 h at 27°C, treated cells were fixed with methanol and subjected to immunostaining and DAPI staining, as described above. The percentage of condensed and/or fragmented nuclei was determined for three independent experiments (of each condition mentioned above), each consisting of two replicate wells. At least 500 nuclei per replicate were counted.

### Detection of apoptotic DNA fragmentation by TUNEL staining

Infected and control cells grown in 24-well plates or chamberslides were fixed with formaldehyde as described for FISH. Cells were then permeabilized in 0.1% Triton-X 100, 0.1% sodium citrate for 10 min and blocked with 2% BSA in PBS for 20 min. Immunostaining of bacteria occurred as described above. The TUNEL reaction was carried out according to the instructions of the *in situ* cell death detection kit (Roche, Austria). In case of the TUNEL positive control, cells were pre-incubated with DNase I (Sigma-Aldrich; 100 U/ml in 50 mM Tris/HCl, 1 mg/ml BSA, pH 7.5) for 10 min. For the negative control, cells were incubated in labelling solution devoid of the enzyme terminal deoxynucleotidyl transferase (TdT). Cells were finally embedded in Mowiol and analyzed at the epifluorescence microscope.

### Detection of caspase activity

Infected and control cells grown in 12-well plates were incubated for indicated periods of time. Culture supernatants were collected and centrifuged (3800× g, 10 min, 4°C) to remove residual cells. Cell lysates were prepared by addition of 150 µl lysis buffer (50 mM HEPES (pH 7.4), 5 mM CHAPS, 5 mM DTT) to each well followed by incubation on ice for 20 min. Lysates were centrifuged (20 800× g, 10 min, 4°C) to remove insoluble cell debris. Samples were stored at −80°C until analysis. Effector caspase activity was measured using a fluorimetric caspase 3 assay kit (Sigma-Aldrich) and a Tecan Infinite M200 plate reader (Tecan, Austria). For each measurement, 5 µl lysate or 20 µl supernatant were applied. Each sample was analyzed in duplicate (technical replicates) and data were collected for at least four replicate infections (biological replicates). Values are presented as arbitrary relative fluorescence units without normalization.

### Statistical analysis

Data obtained for the analysis of nuclear morphology, for monitoring of caspase activity, and for the analysis of the proportion of infected cells during the course of infection, were analyzed using one-way analysis of variance (ANOVA) to test for statistical significant (p≤0.05) differences between groups. Scheffé's post hoc test was then applied to specify groups that significantly (***, p≤0.001; **, p≤0.01; *, p≤0.05) diverged from the untreated control (nuclear morphology) or 0 h p.i. (caspase activity, percentage of infected cells). The unpaired student's t-test was additionally applied to compare the percentage of infected cells between infections that occurred in the absence or presence of Z-VAD-FMK for each time point. All three tests were carried out using the software PASW statistics version 17. In the case of the infection analysis by FISH, infected cells were additionally classified into different groups according to their number of intracellular bacteria. A χ^2^ test was used to compare the distribution of cells among these groups for each time point between infections carried out in absence or presence of Z-VAD-FMK (***, p≤0.001; **, p≤0.01; *, p≤0.05).

## Supporting Information

Figure S1 **Effect of centrifugation and prolonged incubation on the infection efficiency.** Infectious *Pa. acanthamoebae* (MOI 5) were added to S2 cells, followed by centrifugation (15 min at 130× g) if indicated. The growth medium was exchanged either immediately after addition of bacteria (and centrifugation) or after a 2 h incubation period. Infection and all subsequent incubation steps were carried out in medium containing the pan caspase inhibitor Z-VAD-FMK (10 µM). At 46 h p.i. bacteria were detected with the FISH probe UV7-763 (Cy3, red). Representative images are shown in (A). The bar indicates 20 µm. The percentage of infected cells was determined and is depicted in (B). Mean values and standard deviations of 4 replicates are shown. At least 600 cells were examined for each replicate (ANOVA & Scheffé; ***, p≤0.001; *, p≤0.05; ns, not significant).
(TIF)

Figure S2 **Infection cycle of** ***S. negevensis*** **(A) and** ***Pa. acanthamoebae*** **(B) in Sf9 cells.** Sf9 cells were either left untreated or were infected with *S. negevensis* (MOI 5) (A) or *Pa. acanthamoebae* (MOI 1) (B). At indicated time points, bacteria were visualized by immunostaining (green) using antibodies raised against the protochlamydial heat-shock protein DnaK (A), or purified *Pa. acanthamoebae* UV7 (B). DNA was stained with DAPI (blue). The bar corresponds to 10 µm.
(TIF)

Figure S3 **Morphological and nuclear changes in Sf9 cells after infection with** ***Parachlamydiaceae*** **.** Sf9 cells were infected with *Pa. acanthamoebae* or *P. amoebophila* (MOI 2.5). At 10 h p.i. DNA was stained with DAPI (blue). Untreated cells and cells treated with the apoptosis inducer ActD (10 h) are shown for comparison. The bar corresponds to 10 µm.
(TIF)

Figure S4 **Internucleosomal DNA fragmentation in Sf9 cells infected with** ***Parachlamydiaceae*** **.** Insect cells were treated with infectious (Inf), heat-inactivated (Hi), or UV-inactivated (UVi) *P. amoebophila* or *Pa. acanthamoebae* at a MOI of 5, followed by incubation for 24 h. Cells treated with ActD (14 h) served as positive control, and untreated cells (Ut) as negative control. Extracted DNA was separated on 2% agarose gels to visualize apoptotic DNA ladders consisting of bands that are multiplies of about 180–200 bp in size (Method S1). Band sizes of the standard ladder (L) and approximate sizes of apoptotic DNA fragments are given in bp.
(TIF)

Figure S5 **Detection of DNA fragmentation by TUNEL staining in S9 cells infected with** ***Parachlamydiaceae*** **.** Sf9 cells were either left untreated, incubated with ActD (10 h), or infected with *Pa. acanthamoebae* or *P. amoebophila* (MOI 2.5, 10 h). Bacteria were detected by immunostaining using antibodies raised against purified bacteria (red). TUNEL-positive nuclei are shown in green. Two additional controls were included, a negative control where TdT was omitted from the TUNEL reaction mixture and a positive control where cells were preincubated with DNase I to experimentally introduce DNA double strand breaks in all (also non-apoptotic) cells. Note that apart from this control, TUNEL-positive cells typically display other characteristic features of apoptotic cells, such as condensed and fragmented nuclei and formation of apoptotic bodies. After infection, TUNEL-positive cells were also frequently associated with bacteria. The bar indicates 10 µm.
(TIF)

Figure S6 **Time course of** ***Parachlamydiaceae*** **-induced effector caspase activity in Sf9 cells.** Sf9 cells were infected with *Pa. acanthamoebae* (A), *P. amoebophila* (B), or *S. negevensis* at a MOI of 5. Untreated cells (Ut) and cells treated with heat-inactivated bacteria (Hi) or with infectious bacteria in presence of the pan caspase inhibitor Z-VAD-FMK (10 µM) served as controls. Activity of effector caspases in cell lysates (dark gray) and culture supernatants (light gray) was measured at indicated time points by application of an *in vitro* DEVD cleavage assay, in which substrate cleavage results in an increase in fluorescence intensity (FI). Mean values and standard deviations of four replicates are shown. Statistical significant differences compared to 0 h p.i. are indicated (ANOVA & Scheffé ; ***, p≤0.001; **, p≤0.01; *, p≤0.05). ActD-treated cells (14 h) were used as additional positive control for the assay and resulted in mean fluorescence intensities of 2761 and 82 (standard deviation 659 and 36) in cell lysates and supernatants, respectively.
(TIF)

Figure S7 ***Parachlamydiaceae*** **-induced changes in nuclear morphology in Sf9 cells depend on bacterial activity.** Sf9 cells were either left untreated or were treated with SPG buffer, amoebal lysate, infectious *Parachlamydiaceae* (*Pa. acanthamoebae* or *P. amoebophila*; MOI 5) in absence (Inf) or presence of the protein synthesis inhibitor doxycycline (Inf-Dox), heat-inactivated bacteria (Hi), UV- inactivated bacteria (UVi), a sterile-filtrate of the suspension of purified infectious bacteria (Filtrate) or a supernatant collected 48 h p.i. from an infected (MOI 5) apoptotic culture (Supernatant). For comparison, cells treated with infectious *S. negevensis* Z (MOI 5 or 50, as indicated) are shown. After 48 h incubation, DNA was stained with DAPI and the proportion of nuclei with altered morphology was determined. Mean values and standard deviations of six replicates (derived from three independent experiments) are shown. At least 500 nuclei per replicate were considered. Statistically significant differences compared to the untreated cells are indicated (ANOVA & Scheffé; ***, p≤0.001; **, p≤0.01; *, p≤0.05).
(TIF)

Figure S8 **Effect of caspase inhibition on the infection of Sf9 cells with** ***S. negevensis*** **or** ***Pa. acanthamoebae*** **.** Sf9 cells were infected with *S. negevensis* or *Pa. acanthamoebae* (MOI 5) and were incubated for the indicated time periods in the absence or presence of the pan caspase inhibitor Z-VAD-FMK (10 µM). Bacteria were detected by FISH using the probe Chls-0523 in combination with the probe Simneg183 (*S. negevensis*) or UV7-763 (*Pa. acanthamoebae*), respectively. In (A) the percentage of infected cells observed in the absence (blue) or presence (red) of Z-VAD-FMK is shown. Black stars indicate statistically significant differences between both curves (t-test) and colored stars indicate significant differences to the respective 0 h p.i. time point (ANOVA & Scheffé). Numbers of intracellular bacteria per infected cell were determined and are depicted in (B). Infected cells were classified into 5 groups according to the number of intracellular bacteria (1–3, blue; 4–10, rose; 11–30, green; 31–100, orange; >100, red). Stars indicate statistically different distributions among these classes at a given time point between infections carried out in the absence or presence of Z-VAD-FMK (χ^2^ test). In (A) and (B) mean values and standard deviations of three replicates are shown (***, p≤0.001; **, p≤0.01; *, p≤0.05). The gray boxes indicate time points that were analyzed after cells had been passaged.
(TIF)

Figure S9 **Transmission electron micrographs of S2 cells infected with** ***Parachlamydiaceae*** **in presence or absence of Z-VAD-FMK.** S2 cells were infected with *Pa. acanthamoebae* or *P. amoebophila* (MOI 5) and were incubated for 48 h in the absence or presence of the pan caspase inhibitor Z-VAD-FMK (20 µM) before electron microscopic examination (Method S2). Representative images of secondary necrotic cells (observed in the absence of caspase inhibition) and intact infected cells (observed in presence of the caspase inhibitor) are shown. The bar indicates 2 µm.
(TIF)

Method S1 **Detection of internucleosomal DNA fragmentation on agarose gels.**
(DOC)

Method S2 **Transmission electron microscopy.**
(DOC)
