# Supplementary figures and images for: Lack of Effective Anti-Apoptotic Activities Restricts Growth of Parachlamydiaceae in Insect Cells
Source: PLoS One. 2012 Jan 9;7(1):e29565. doi: 10.1371/journal.pone.0029565 (PMC3253803; doi:10.1371/journal.pone.0029565)

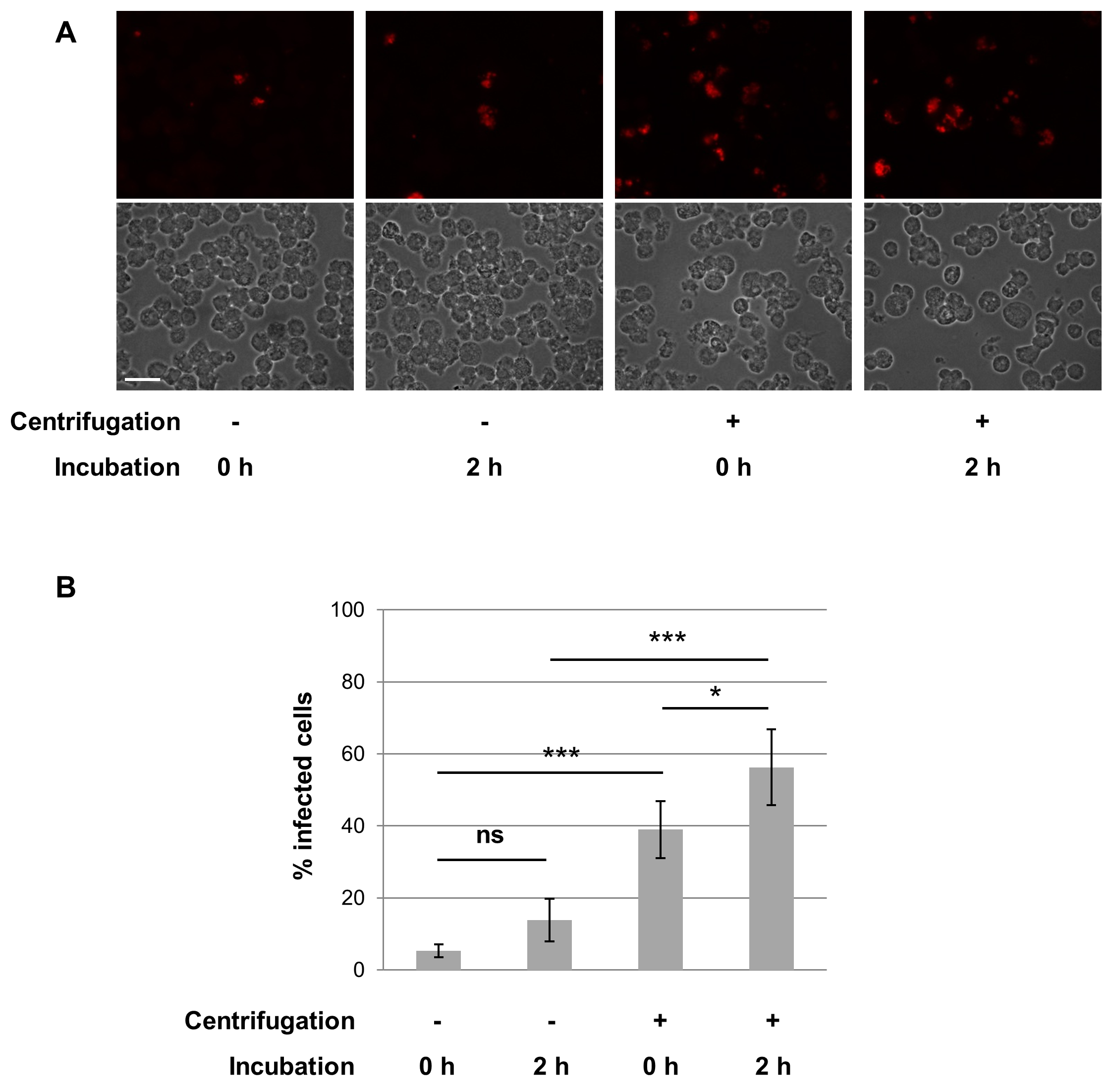

Supplement: Figure S1 — Effect of centrifugation and prolonged incubation on the infection efficiency. Infectious Pa. acanthamoebae (MOI 5) were added to S2 cells, followed by centrifugation (15 min at 130× g) if indicated. The growth medium was exchanged either immediately after addition of bacteria (and centrifugation) or after a 2 h incubation period. Infection and all subsequent incubation steps were carried out in medium containing the pan caspase inhibitor Z-VAD-FMK (10 µM). At 46 h p.i. bacteria were detected with the FISH probe UV7-763 (Cy3, red). Representative images are shown in (A). The bar indicates 20 µm. The percentage of infected cells was determined and is depicted in (B). Mean values and standard deviations of 4 replicates are shown. At least 600 cells were examined for each replicate (ANOVA & Scheffé; ***, p≤0.001; *, p≤0.05; ns, not significant). (TIF) [file pone.0029565.s001.tif]

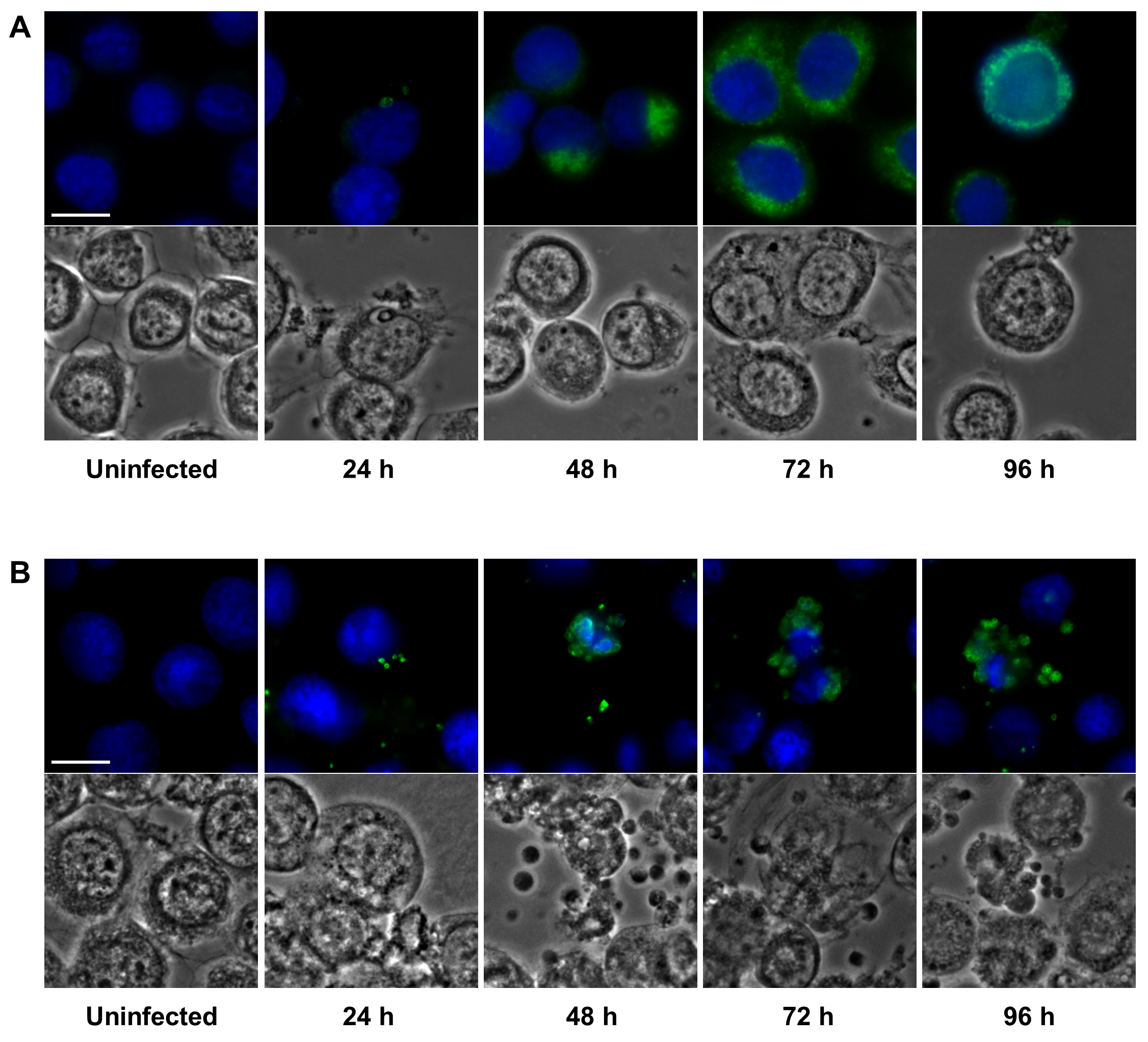

Supplement: Figure S2 — Infection cycle of S. negevensis (A) and Pa. acanthamoebae (B) in Sf9 cells. Sf9 cells were either left untreated or were infected with S. negevensis (MOI 5) (A) or Pa. acanthamoebae (MOI 1) (B). At indicated time points, bacteria were visualized by immunostaining (green) using antibodies raised against the protochlamydial heat-shock protein DnaK (A), or purified Pa. acanthamoebae UV7 (B). DNA was stained with DAPI (blue). The bar corresponds to 10 µm. (TIF) [file pone.0029565.s002.tif]

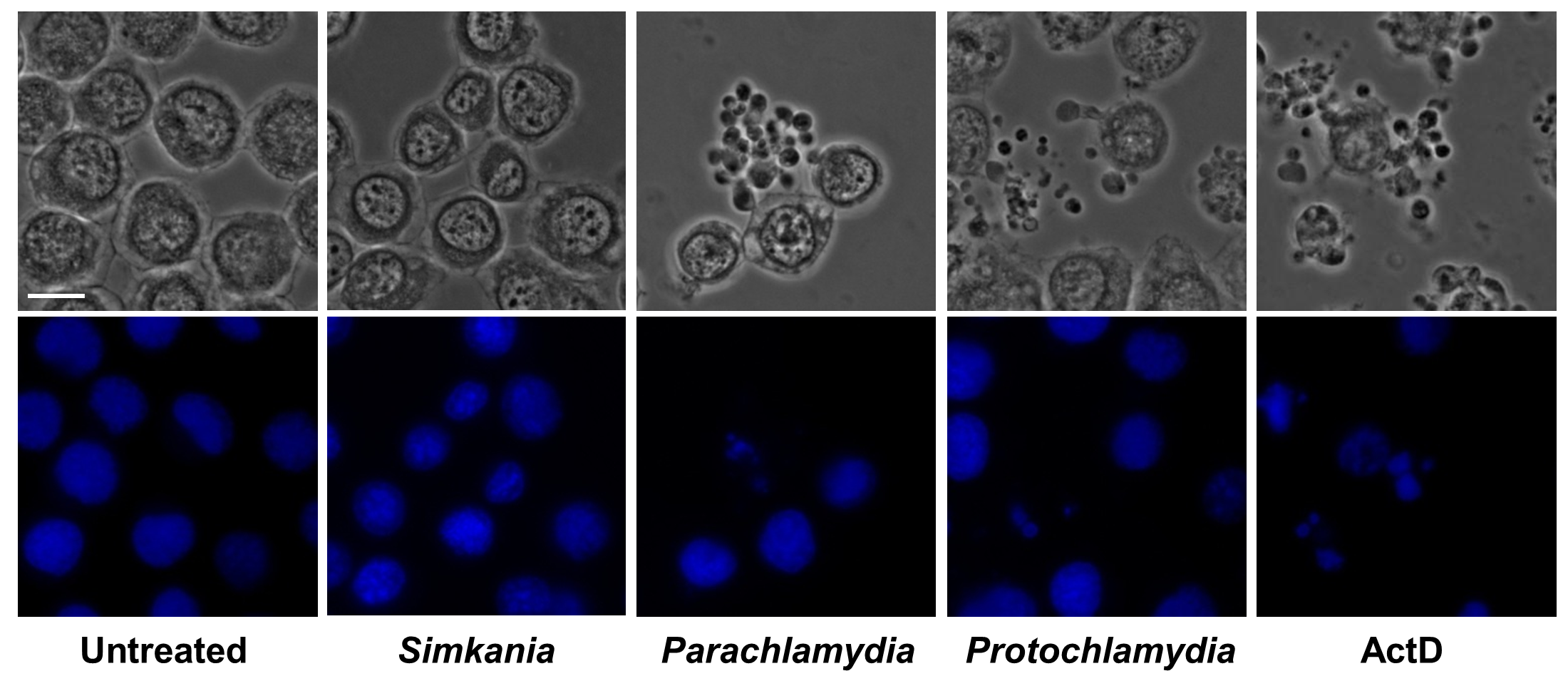

Supplement: Figure S3 — Morphological and nuclear changes in Sf9 cells after infection with Parachlamydiaceae . Sf9 cells were infected with Pa. acanthamoebae or P. amoebophila (MOI 2.5). At 10 h p.i. DNA was stained with DAPI (blue). Untreated cells and cells treated with the apoptosis inducer ActD (10 h) are shown for comparison. The bar corresponds to 10 µm. (TIF) [file pone.0029565.s003.tif]

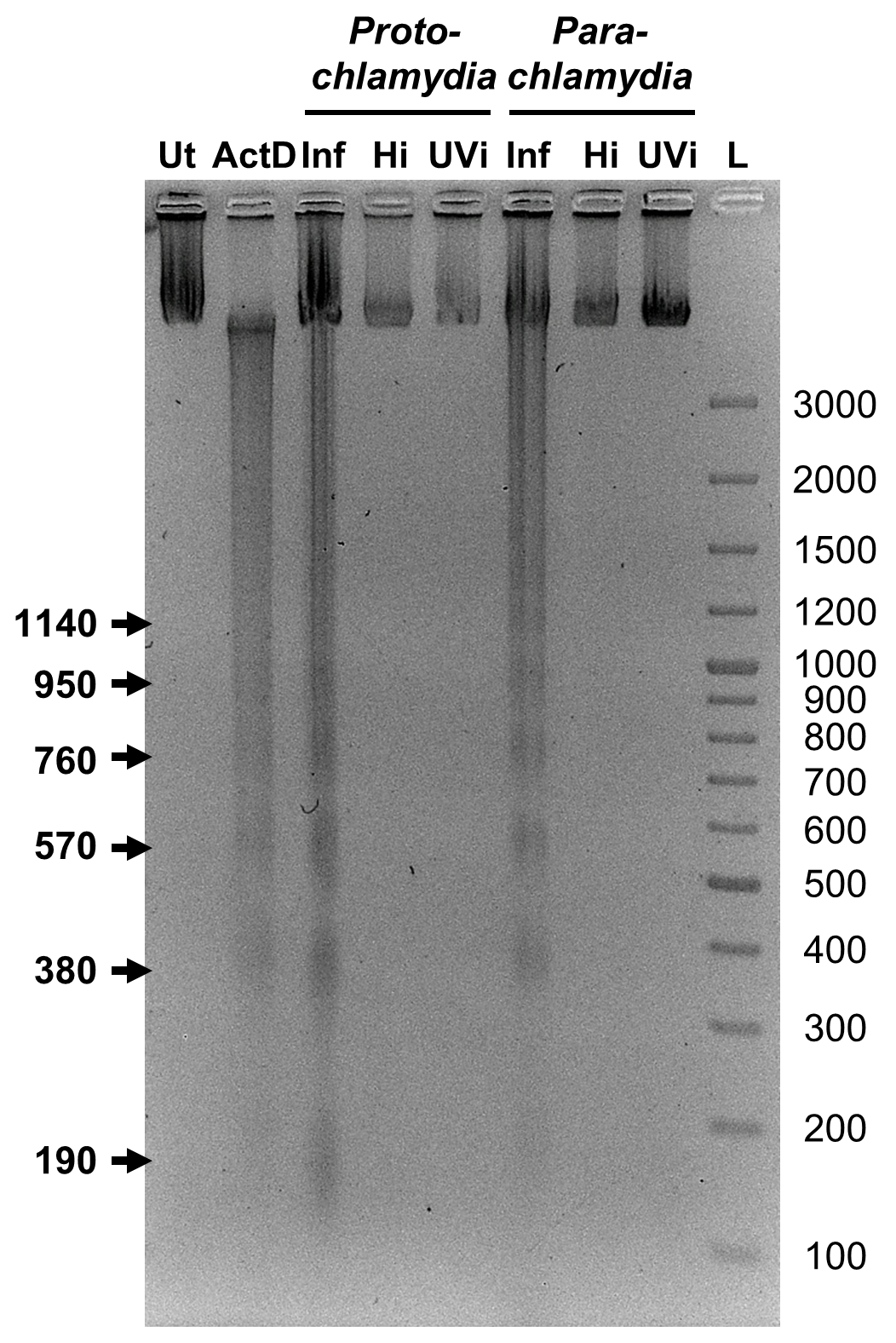

Supplement: Figure S4 — Internucleosomal DNA fragmentation in Sf9 cells infected with Parachlamydiaceae . Insect cells were treated with infectious (Inf), heat-inactivated (Hi), or UV-inactivated (UVi) P. amoebophila or Pa. acanthamoebae at a MOI of 5, followed by incubation for 24 h. Cells treated with ActD (14 h) served as positive control, and untreated cells (Ut) as negative control. Extracted DNA was separated on 2% agarose gels to visualize apoptotic DNA ladders consisting of bands that are multiplies of about 180–200 bp in size (Method S1). Band sizes of the standard ladder (L) and approximate sizes of apoptotic DNA fragments are given in bp. (TIF) [file pone.0029565.s004.tif]

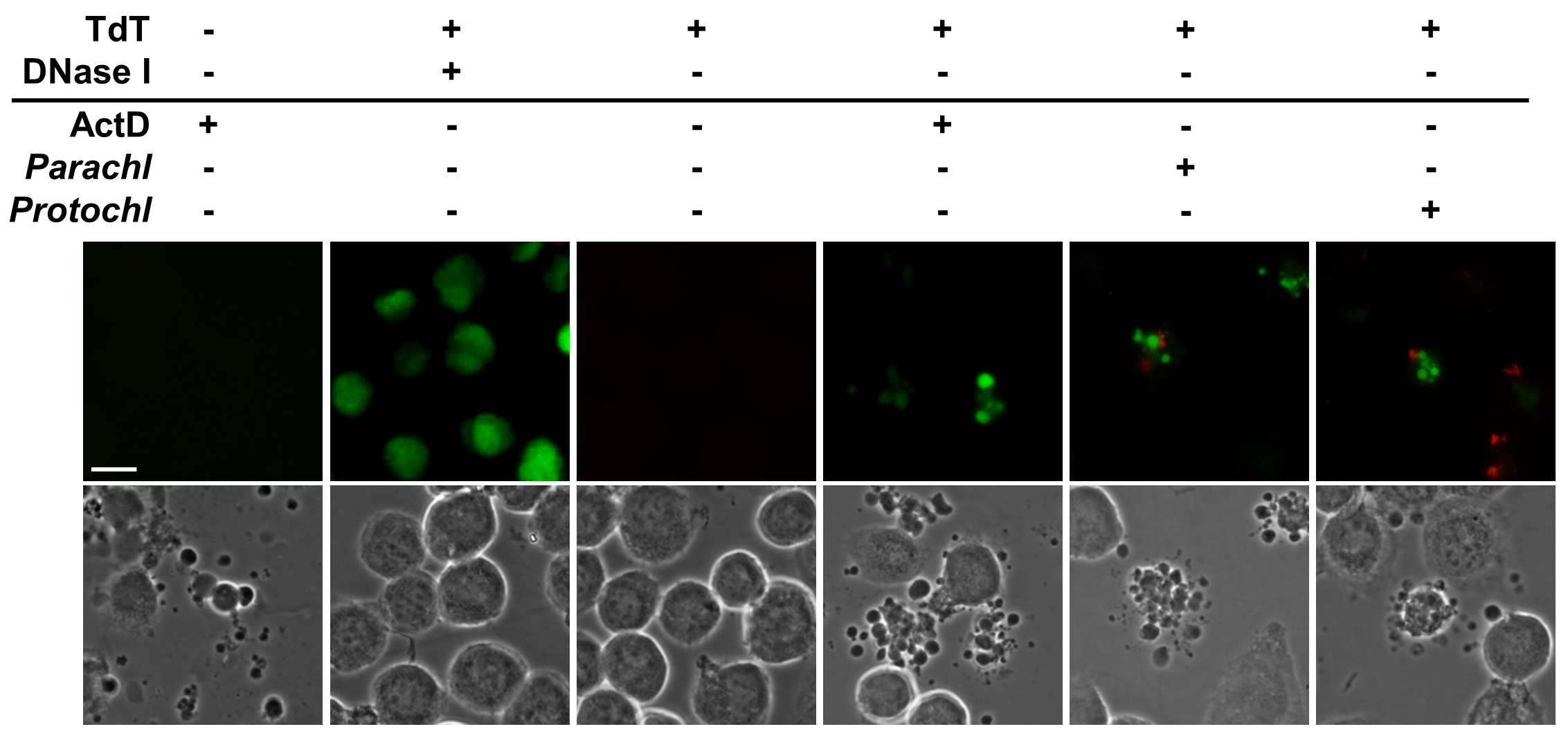

Supplement: Figure S5 — Detection of DNA fragmentation by TUNEL staining in S9 cells infected with Parachlamydiaceae . Sf9 cells were either left untreated, incubated with ActD (10 h), or infected with Pa. acanthamoebae or P. amoebophila (MOI 2.5, 10 h). Bacteria were detected by immunostaining using antibodies raised against purified bacteria (red). TUNEL-positive nuclei are shown in green. Two additional controls were included, a negative control where TdT was omitted from the TUNEL reaction mixture and a positive control where cells were preincubated with DNase I to experimentally introduce DNA double strand breaks in all (also non-apoptotic) cells. Note that apart from this control, TUNEL-positive cells typically display other characteristic features of apoptotic cells, such as condensed and fragmented nuclei and formation of apoptotic bodies. After infection, TUNEL-positive cells were also frequently associated with bacteria. The bar indicates 10 µm. (TIF) [file pone.0029565.s005.tif]

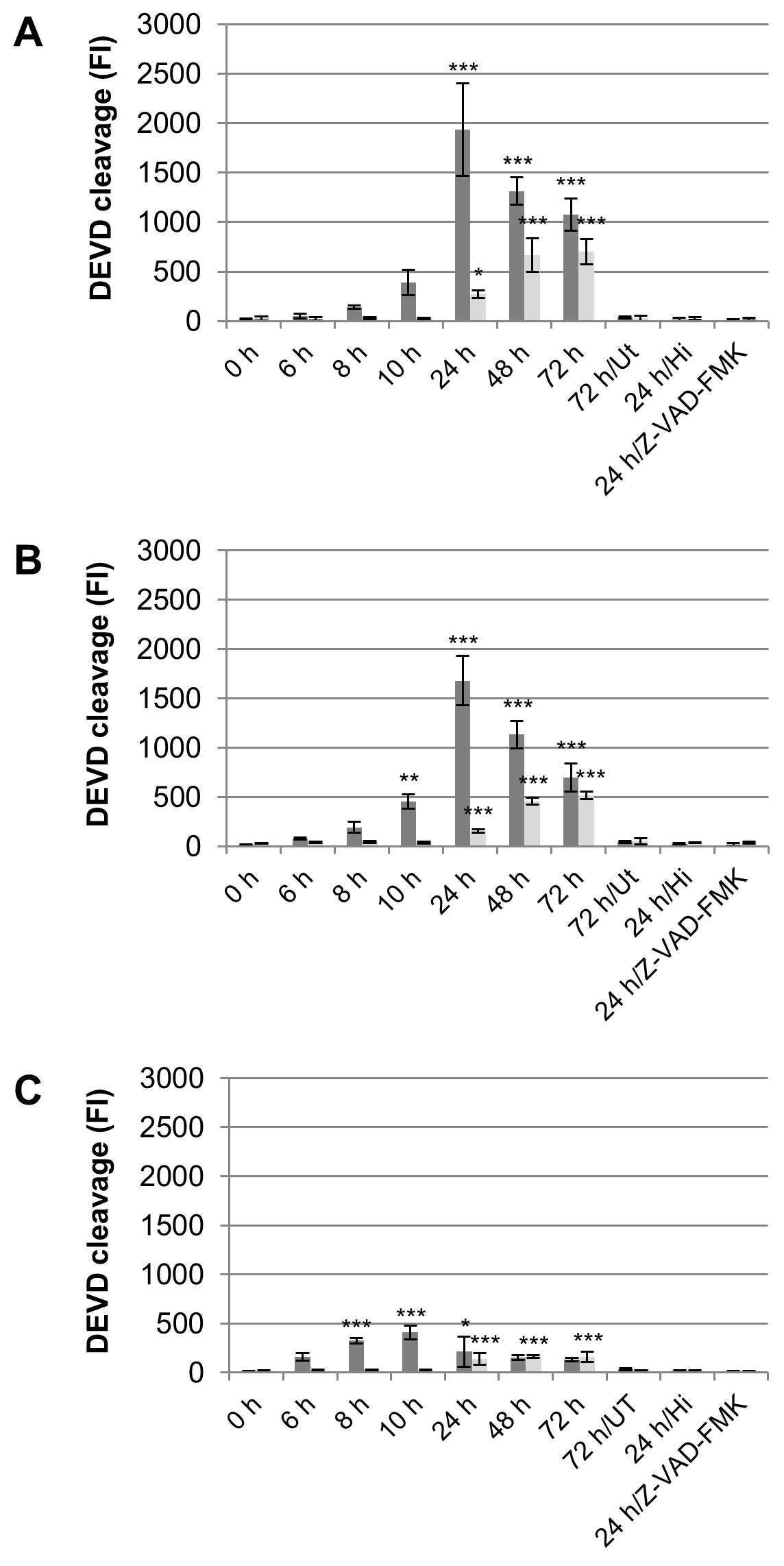

Supplement: Figure S6 — Time course of Parachlamydiaceae -induced effector caspase activity in Sf9 cells. Sf9 cells were infected with Pa. acanthamoebae (A), P. amoebophila (B), or S. negevensis at a MOI of 5. Untreated cells (Ut) and cells treated with heat-inactivated bacteria (Hi) or with infectious bacteria in presence of the pan caspase inhibitor Z-VAD-FMK (10 µM) served as controls. Activity of effector caspases in cell lysates (dark gray) and culture supernatants (light gray) was measured at indicated time points by application of an in vitro DEVD cleavage assay, in which substrate cleavage results in an increase in fluorescence intensity (FI). Mean values and standard deviations of four replicates are shown. Statistical significant differences compared to 0 h p.i. are indicated (ANOVA & Scheffé ; ***, p≤0.001; **, p≤0.01; *, p≤0.05). ActD-treated cells (14 h) were used as additional positive control for the assay and resulted in mean fluorescence intensities of 2761 and 82 (standard deviation 659 and 36) in cell lysates and supernatants, respectively. (TIF) [file pone.0029565.s006.tif]

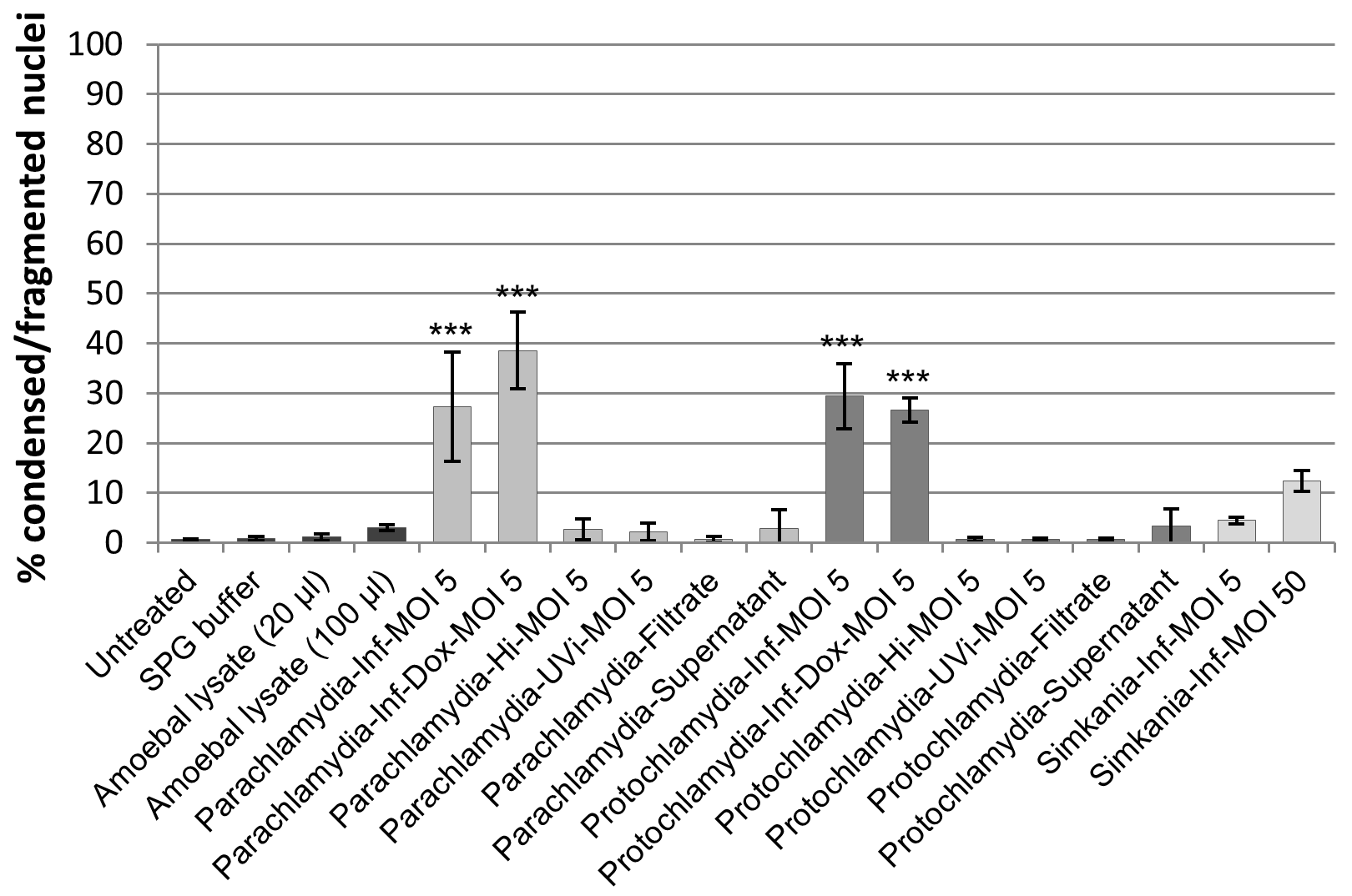

Supplement: Figure S7 — Parachlamydiaceae -induced changes in nuclear morphology in Sf9 cells depend on bacterial activity. Sf9 cells were either left untreated or were treated with SPG buffer, amoebal lysate, infectious Parachlamydiaceae (Pa. acanthamoebae or P. amoebophila; MOI 5) in absence (Inf) or presence of the protein synthesis inhibitor doxycycline (Inf-Dox), heat-inactivated bacteria (Hi), UV- inactivated bacteria (UVi), a sterile-filtrate of the suspension of purified infectious bacteria (Filtrate) or a supernatant collected 48 h p.i. from an infected (MOI 5) apoptotic culture (Supernatant). For comparison, cells treated with infectious S. negevensis Z (MOI 5 or 50, as indicated) are shown. After 48 h incubation, DNA was stained with DAPI and the proportion of nuclei with altered morphology was determined. Mean values and standard deviations of six replicates (derived from three independent experiments) are shown. At least 500 nuclei per replicate were considered. Statistically significant differences compared to the untreated cells are indicated (ANOVA & Scheffé; ***, p≤0.001; **, p≤0.01; *, p≤0.05). (TIF) [file pone.0029565.s007.tif]

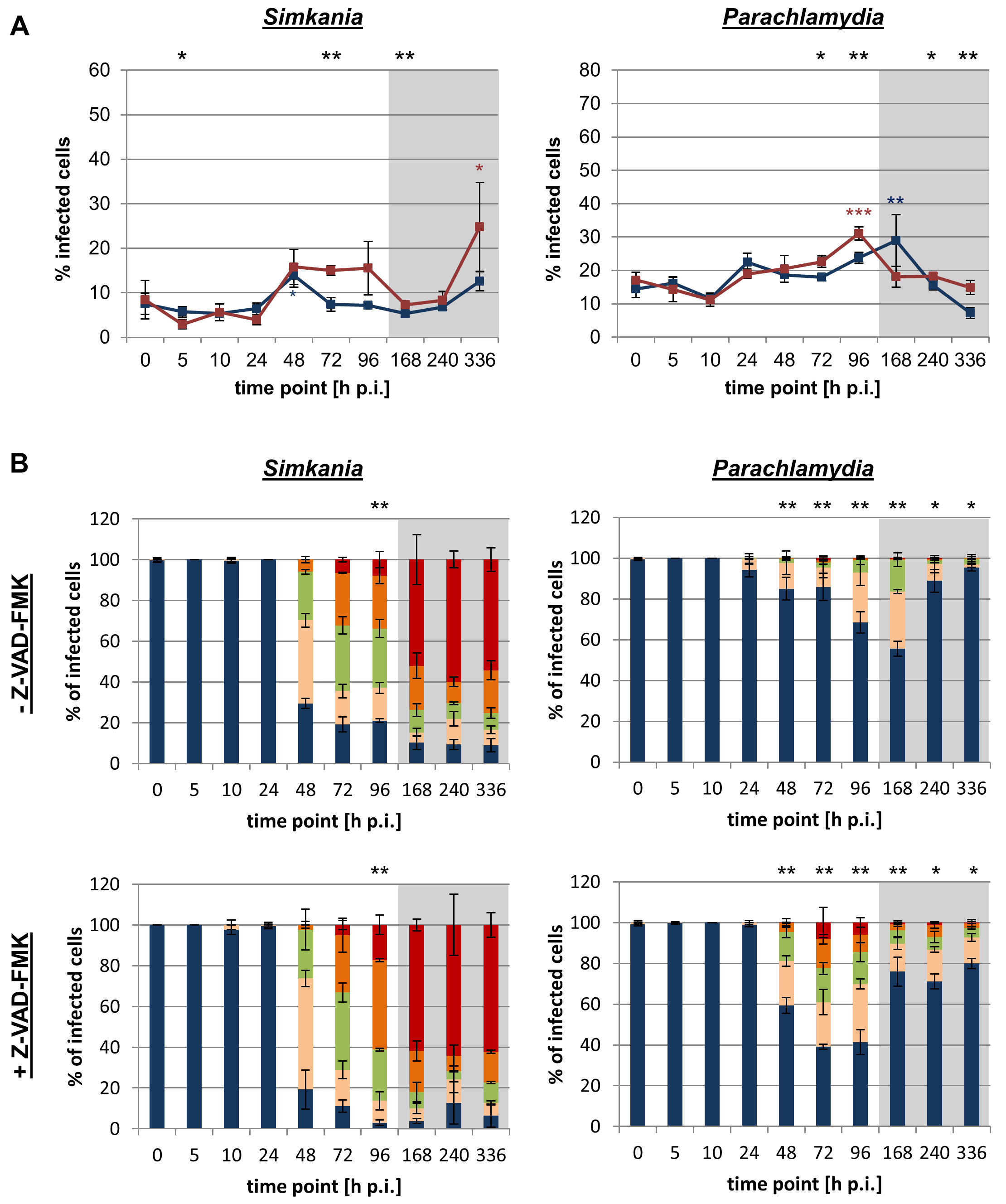

Supplement: Figure S8 — Effect of caspase inhibition on the infection of Sf9 cells with S. negevensis or Pa. acanthamoebae . Sf9 cells were infected with S. negevensis or Pa. acanthamoebae (MOI 5) and were incubated for the indicated time periods in the absence or presence of the pan caspase inhibitor Z-VAD-FMK (10 µM). Bacteria were detected by FISH using the probe Chls-0523 in combination with the probe Simneg183 (S. negevensis) or UV7-763 (Pa. acanthamoebae), respectively. In (A) the percentage of infected cells observed in the absence (blue) or presence (red) of Z-VAD-FMK is shown. Black stars indicate statistically significant differences between both curves (t-test) and colored stars indicate significant differences to the respective 0 h p.i. time point (ANOVA & Scheffé). Numbers of intracellular bacteria per infected cell were determined and are depicted in (B). Infected cells were classified into 5 groups according to the number of intracellular bacteria (1–3, blue; 4–10, rose; 11–30, green; 31–100, orange; >100, red). Stars indicate statistically different distributions among these classes at a given time point between infections carried out in the absence or presence of Z-VAD-FMK (χ2 test). In (A) and (B) mean values and standard deviations of three replicates are shown (***, p≤0.001; **, p≤0.01; *, p≤0.05). The gray boxes indicate time points that were analyzed after cells had been passaged. (TIF) [file pone.0029565.s008.tif]

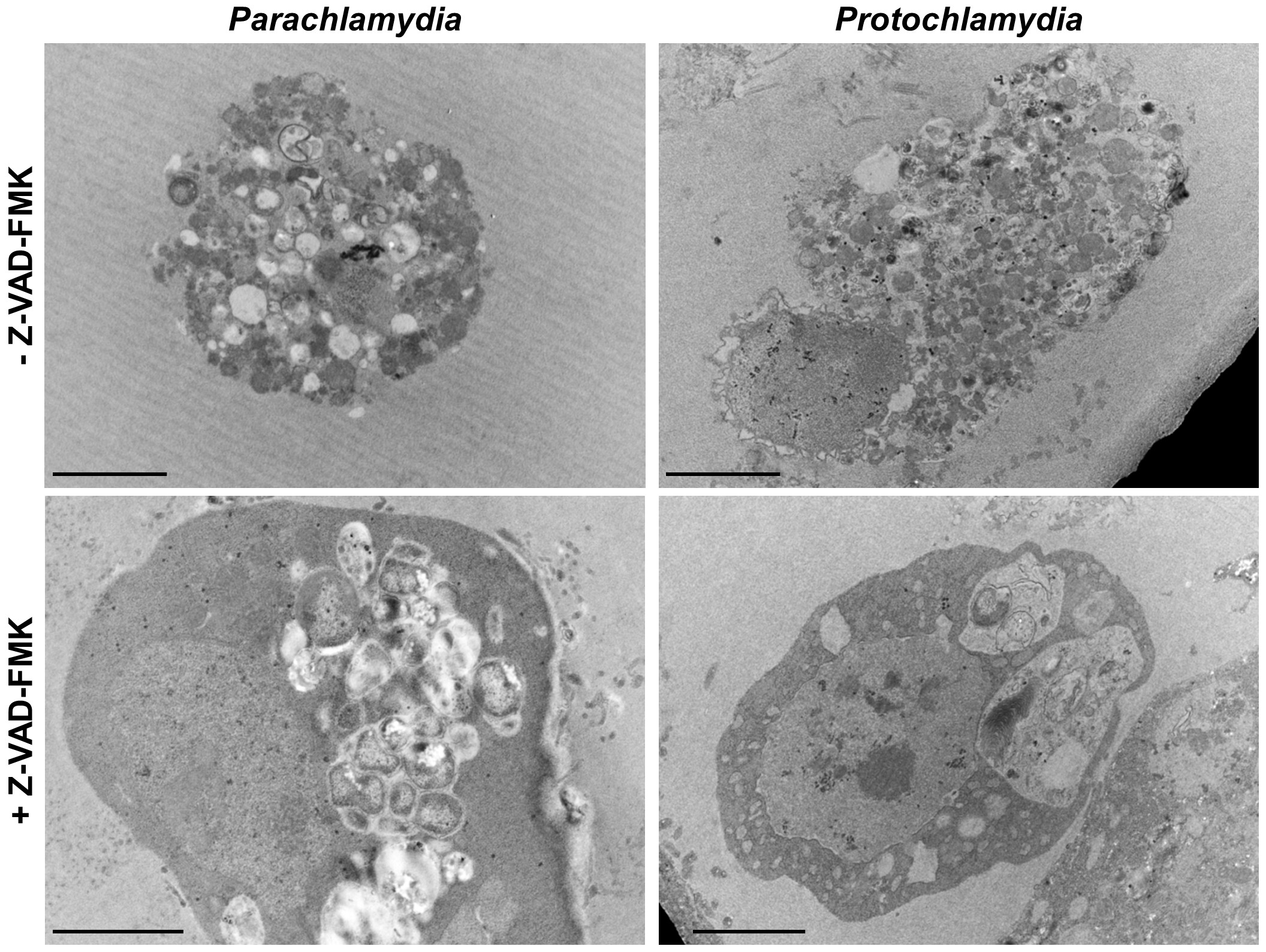

Supplement: Figure S9 — Transmission electron micrographs of S2 cells infected with Parachlamydiaceae in presence or absence of Z-VAD-FMK. S2 cells were infected with Pa. acanthamoebae or P. amoebophila (MOI 5) and were incubated for 48 h in the absence or presence of the pan caspase inhibitor Z-VAD-FMK (20 µM) before electron microscopic examination (Method S2). Representative images of secondary necrotic cells (observed in the absence of caspase inhibition) and intact infected cells (observed in presence of the caspase inhibitor) are shown. The bar indicates 2 µm. (TIF) [file pone.0029565.s009.tif]
