# Supplementary material for: Lack of Effective Anti-Apoptotic Activities Restricts Growth of Parachlamydiaceae in Insect Cells
Source: PLoS One. 2012 Jan 9;7(1):e29565. doi: 10.1371/journal.pone.0029565 (PMC3253803; doi:10.1371/journal.pone.0029565)
Supplement: Method S1 — Detection of internucleosomal DNA fragmentation on agarose gels. (DOC) [file pone.0029565.s010.doc]

**Method S1: Detection of internucleosomal DNA fragmentation on agarose gels**

Infected and control cells grown in culture flasks were harvested by centrifugation (1700 x g, 10 min, 4°C) and washed twice with PBS. Pellets were resuspended in 400 µl lysis buffer (100 mM NaCl, 10 mM Tris/HCl pH 7.9, 25 mM EDTA, 0.5% SDS, 1 mg/ml RNase A) and incubated for 1 h at 37°C. Proteinase K (0.3 mg/ml) was added, followed by overnight incubation at 55°C. Then, 1.5 volumes phenol:chloroform:isoamyl alcohol (25:24:1) were added and tubes were inverted several times followed by centrifugation (15 100 x g, 15 min, 4°C). The watery phase was transferred to a fresh tube and the extraction was repeated twice with chloroform:isoamyl alcohol (24:1). DNA was precipitated by addition of two volumes of ice-cold ethanol and 0.1 volumes 3 M sodium acetate, followed by incubation at -80°C for 1 h and centrifugation (as above). The DNA was washed with 70% ethanol, resuspended in a small volume of 10 mM Tris/HCl (pH 8.0), 1 mM EDTA, and quantified using a NanoDrop ND-1000 spectrophotometer (PeqLab, Germany). A total of 3 µg (Aa23 cells) or 4 µg (Sf9 cells) DNA per sample was separated on a 2% agarose gel and visualized with ethidium bromide. The GeneRulerTM 100 bp DNA ladder plus (Fermentas, Germany) was used as size marker.
