# Supplementary material for: Lack of Effective Anti-Apoptotic Activities Restricts Growth of Parachlamydiaceae in Insect Cells
Source: PLoS One. 2012 Jan 9;7(1):e29565. doi: 10.1371/journal.pone.0029565 (PMC3253803; doi:10.1371/journal.pone.0029565)
Supplement: Method S2 — Transmission electron microscopy. (DOC) [file pone.0029565.s011.doc]

**Method S2: Transmission electron microscopy**

S2 cells grown in culture flasks were infected with *Pa. acanthamoebae* UV7 or *P. amoebophila* UWE25 (MOI 5) and incubated in presence or absence of the pan-caspase inhibitor Z-VAD-FMK (20 µM; Promega, USA). At 48 h p.i. cells were collected (7200 x g, 10 min), fixed for 1 h at room temperature with 2.5% glutaraldehyde in 0.1 M sodium cacodylate buffer (pH 6.4) containing 0.1 M sucrose, and washed three times for 10 min with 0.1 M cacodylate, 0.1 M sucrose (pH 6.4). Cells were then resuspended in 100 µl of pre-warmed 12% buffered gelatin (Dr. Oetker, Germany) and incubated at 37°C for 10 min. Cell-gelatin mixtures were pelleted (230 x g, 5 min), cooled on ice for 1 h, and cross-linked for 1 h on ice with fixative solution. Gelatin blocks were then cut into 1 mm thick pieces that were washed as described above. Secondary fixation was conducted in 1% buffered osmium tetroxide for 90 min at room temperature, followed by dehydration through a graded ethanol series and infiltration with acetone. Infiltration with Low Viscosity Resin (Agar Scientific, UK) occurred for 2 h in a 3:1 acetone-resin mixture and 4 h in a 1:1 mixture, before residual solvent was allowed to evaporate overnight. The pure resin was replaced three times within 6 h before final embedding. Resin polymerization occurred at 60°C for 24 h. Ultrathin sections were prepared and contrasted with 2% uranyl acetate and 0.5% lead citrate before electron microscopic examination (Philips EM 208, Koninklijke Philips Electronics N.V, The Netherlands).
